# Supplementary material for: Phenotypes of non-alcoholic fatty liver disease (NAFLD) and all-cause mortality: unsupervised machine learning analysis of NHANES III
Source: BMJ Open. 2022 Nov 23;12(11):e067203. doi: 10.1136/bmjopen-2022-067203 (PMC9685238; doi:10.1136/bmjopen-2022-067203)
Supplement: Supplementary data [file bmjopen-2022-067203supp001.pdf]

**SUPPLEMENTARY MATERIALS**

**Phenotypes of non-alcoholic fatty liver disease (NAFLD) and all-cause mortality: Unsupervised machine learning analysis with NHANES III**

Table of Contents

Supplementary Methods ........ 2

Data preparation ........ 2

Study sample ........ 2

Number of clusters ........ 3

Supplementary Table 1. Pair-wise comparisons between clusters. ........ 7

Supplementary Figure 1. Survival curve by cluster. ........ 8

Supplementary Figure 2. Schoenfeld residuals for the crude Cox-proportional hazard model. ........ 9



Carrillo-Larco RM, *et al. BMJ Open* 2022; 12:e067203. doi: 10.1136/bmjopen-2022-067203

**Supplementary Methods**

All the analyses were conducted with R and Python. The codes, either R scripts or Python Jupyter notebooks, are available as supplementary materials together with this manuscript.

**Data preparation**

We used individual-level data from the National Health and Nutrition Examination Survey (NHANES) III conducted between 1988-1994. All datasets were downloaded from the NHANES website on March 3rd, 2022; the mortality dataset was download on May 27th, 2022. The authors did not have privileged access. Data were pre-processed with R (NHANESiii_extraction.R). We used these datasets: adult.dat.txt (n=20,050); lab.sas.txt (n=29,314); exam.sas.txt (n=31,311); examdr.sas.txt (n=30,818); and HGUHS (n=14,797). The adult dataset was used as the prime; that is, the other datasets were merged to the adult dataset: adult + lab (n=20,050); + exam (n=20,050); + examdr (n=20,050); + HGUHS (n=20,050). We kept only the variables of interest, checked each variable, and recoded them as needed; for example, 9999 or equivalent values were set to missing, and 2 were coded to 0 when referred to ‘no’. We merged the mortality dataset (n=33,994) with the pooled dataset and the sample size was kept at 20,050 (i.e., the pooled dataset was used as the prime) with 79 variables.

**Study sample**

We followed these criteria to select the study sample (Flowchart 1). First, we only included people in the age range from 20 to 74 (inclusive) years. Second, we only kept observations with hepatic imaging data. Third, we excluded people with positive evidence of Hepatitis B (HBsAG) or Hepatitis C (antiHCV). Fourth, we excluded people

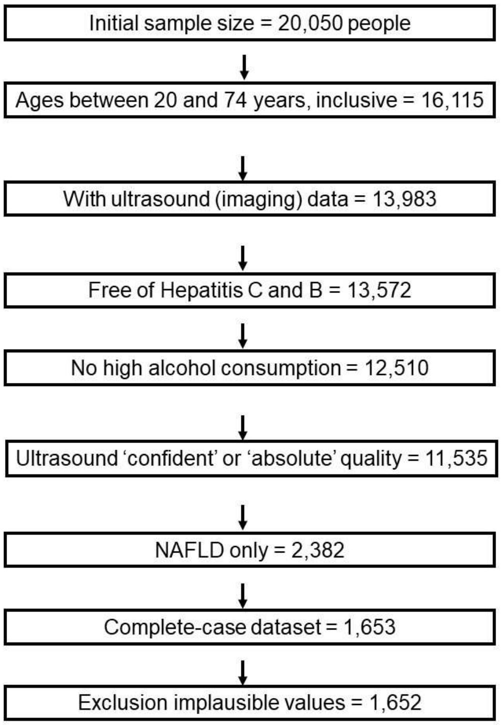


**Flowchart 1. Study population.**



Carrillo-Larco RM, *et al. BMJ Open* 2022; 12:e067203. doi: 10.1136/bmjopen-2022-067203

with high alcohol consumption. We used two questions to define high alcohol consumption: i) *In the past 12 months, how many days of the year did you drink any alcoholic beverages?* and ii) *On the average, on the days that you drank alcohol, how many drinks did you have a day?* The answers to the second question were divided by 365.25 to compute the average number of drinks per day, which was then multiplied by the first question (how many days they drunk alcohol) to compute the number of daily alcohol drinks in the last year. For example, a person who drank alcoholic beverages 52 days in the last year, and on each occasion they drank three beverages, their daily consumption would be 52 x 3/365.25 = 0.43 alcoholic beverages per day in the last year. Men with more than two and women with more than one alcoholic beverages per day in the last year were excluded. Fifth, we only included observations which the hepatic imaging was deemed 'confident' or 'absolute'; this, to secure the highest quality of the outcome of interest (NAFLD); in addition, we only include people whose hepatic imaging revealed hepatic steatosis 'moderate-severe'. Sixth, we only kept the 10 predictors of interest and dropped all missing observations; in other words, we were to conduct a complete-case analysis. Seventh, we excluded observations outside the following plausibility ranges to secure high-quality data: BMI below and above 10 kg/m$^2$ and 80 kg/m$^2$; waist circumference below and above 30 cm and 200 cm; systolic blood pressure below and above 70 mmHg an 270 mmHg; fasting plasma glucose below and above 45 mg/dL and 540 mg/dL; total cholesterol below and above 20 mg/dL and 773 mg/dL; and triglycerides below and above 17 mg/dL and 1771 mg/dL. Finally, we included 1,652 observations in the analysis. For further details about this selection process please refer to the Jupyter notebook 1.Cleaning_data.ipynb.

**Number of clusters**

Selecting the *ideal* number of clusters in an unsupervised machine learning model is informed by both the data and expert knowledge. In here, we describe in detail the process we followed to reach the final number of clusters used in the analysis. Details about the analytical steps are presented in the Jupyter notebooks presented along the paper (2.Number_clusters.ipynb).

First, we displayed a dendrogram with Euclidean distances; this plot suggested there were five clusters. Of note, two of the six clusters grouped (very) few observations. Second, we displayed the Elbow plot (Figure 1) for one through 10 clusters. The ideal number of clusters would be that after which the Cost function does not change substantially and is the smallest. The table below shows the Cost function for each number of clusters, and the absolute arithmetic difference between two consecutive Cost functions. According to these figures (Table 1), the optimal number of clusters could be between five and six clusters; moreover, five clusters appeared to be *ideal* because the cost function was smallest and almost constant (~1.4) thereafter.

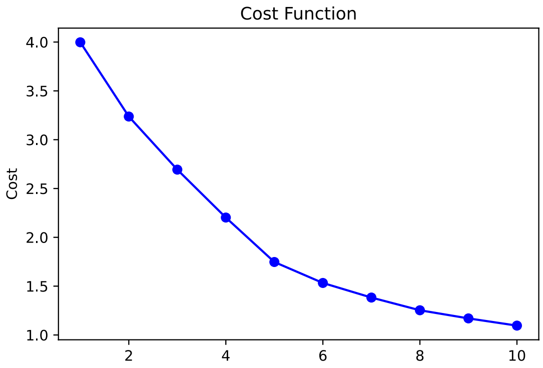

**Figure 2. Elbow plot.**

**Table 1. Cost function for each number of cluster (i.e., elbow plot)**

| Number of clusters | Cost function | Difference with the immediate before |
|---|---|---|
| 1 | 3.9975786924939500 | |
| 2 | 3.2371647353902400 | -0.76 |
| 3 | 2.6931986798721200 | -0.54 |
| 4 | 2.2026180284390100 | -0.49 |
| 5 | 1.7472952094553600 | -0.46 |
| 6 | 1.5326884453790900 | -0.21 |
| 7 | 1.3831729448463400 | -0.15 |
| 8 | 1.2530783891239300 | -0.13 |
| 9 | 1.1698248951603600 | -0.08 |
| 10 | 1.0958516180666800 | -0.07 |

Third, we displayed the Silhouette plots and computed the Silhouette scores between two and 10 clusters (Table below). The number of clusters which Silhouette score is closest to 1 is preferred. According to these



Carrillo-Larco RM, *et al. BMJ Open* 2022; 12:e067203. doi: 10.1136/bmjopen-2022-067203

figures (Table 2), two clusters would be *ideal* (i.e., highest Silhouette score), closely followed by three and four clusters.

**Table 2. Silhouette score for each number of clusters.**

| Number of clusters | Silhouette scores |
|---|---|
| 2 | 0.54608205593638 |
| 3 | 0.49123277407391 |
| 4 | 0.47822718686785 |
| 5 | 0.29615494816696 |
| 6 | 0.30743082083024 |
| 7 | 0.24963726715962 |
| 8 | 0.26441204421507 |
| 9 | 0.25048383421862 |
| 10 | 0.25036003409133 |

Fourth, we calculated the Jaccard index for three, four and five clusters (Table 3). So far, three, four and five clusters appear to be the best options. Jaccard scores ≥0.80 suggest good reproducibility of the cluster and would thus be preferred. According to these figures (Table below), three clusters had the highest Jaccard scores, all of which were ≥0.88. The Jaccard analysis was conducted in R (2.1.Jaccard.R).

**Table 3. Jaccard score for each number of clusters from three to five clusters.**

| | **1 cluster** | **2 clusters** | **3 clusters** | | |
|---|---|---|---|---|---|
| Jaccard bootstrap | 0.8853153 | 0.9666082 | 0.9000518 | | |
| Dissolved | 284 | 0 | 309 | | |
| Recovered | 4402 | 4826 | 4579 | | |
| | **1 cluster** | **2 clusters** | **3 clusters** | **4 clusters** | |
| Jaccard bootstrap | 0.5858137 | 0.8741936 | 0.5433940 | 0.6849515 | |

| | | | | | |
|---|---|---|---|---|---|
| Dissolved | 2564 | 0 | 2801 | 720 | |
| Recovered | 1728 | 4705 | 1279 | 1691 | |
| | **1 cluster** | **2 clusters** | **3 clusters** | **4 clusters** | **5 clusters** |
| Jaccard bootstrap | 0.7489208 | 0.7477933 | 0.5354848 | 0.5248099 | 0.6352783 |
| Dissolved | 1244 | 17 | 2839 | 2834 | 1918 |
| Recovered | 2942 | 2231 | 915 | 1139 | 1905 |

Fifth, exploratory analyses with five and six clusters showed that some clusters had (very) few observations and there were not unique profiles to characterize these clusters. Having four clusters was later discarded because of the Jaccard scores (see Table above). In conclusion, we chose three clusters as the *ideal* number of clusters for the analysis, which is also supported by the Jaccard score and (rather) consistent with the Elbow plot (Figure 1) and the Silhouette score (Table 2).

Of note, in exploratory analyses, we also tried selecting the number of clusters based on other algorithms and the results were consistent with those herein summarized; the algorithms we tried were: Balanced Iterative Reducing and Clustering using Hierarchies (BIRCH); spectral; and agglomerative.



Carrillo-Larco RM, *et al. BMJ Open* 2022; 12:e067203. doi: 10.1136/bmjopen-2022-067203

**Supplementary Table 1. Pair-wise comparisons between clusters.**

| Predictor | Grupo 1 | Grupo 2 | Sample in Group 1 | Sample in Group 2 | Statistic | Degrees of freedom | p-value | Bonferroni-adjusted p-value |
|---|---|---|---|---|---|---|---|---|
| Age | Average | Lipid-Liver | 1376 | 171 | -0.4341 | 228.8684 | 6.650E-01 | 1.000E+00 |
| | Average | Anthro-BP-Glucose | 1376 | 105 | -6.9071 | 132.8967 | 0.000E+00 | 0.000E+00 |
| | Lipid-Liver | Anthro-BP-Glucose | 171 | 105 | -5.1494 | 243.0031 | 0.000E+00 | 0.000E+00 |
| Body Mass Index | Average | Lipid-Liver | 1376 | 171 | -0.8983 | 254.9349 | 3.700E-01 | 1.000E+00 |
| | Average | Anthro-BP-Glucose | 1376 | 105 | -2.7146 | 118.85 | 8.000E-03 | 2.400E-02 |
| | Lipid-Liver | Anthro-BP-Glucose | 171 | 105 | -1.9792 | 167.1476 | 4.900E-02 | 1.470E-01 |
| Waist Circumference | Average | Lipid-Liver | 1376 | 171 | -3.3914 | 256.2415 | 8.000E-04 | 2.400E-03 |
| | Average | Anthro-BP-Glucose | 1376 | 105 | -4.3361 | 125.4833 | 0.000E+00 | 1.000E-04 |
| | Lipid-Liver | Anthro-BP-Glucose | 171 | 105 | -1.7477 | 190.4266 | 8.200E-02 | 2.460E-01 |
| Systolic Blood Pressure | Average | Lipid-Liver | 1376 | 171 | -3.0838 | 221.5132 | 2.000E-03 | 6.000E-03 |
| | Average | Anthro-BP-Glucose | 1376 | 105 | -4.0781 | 120.6513 | 1.000E-04 | 2.000E-04 |
| | Lipid-Liver | Anthro-BP-Glucose | 171 | 105 | -1.4565 | 209.4584 | 1.470E-01 | 4.410E-01 |
| Plasma Glucose | Average | Lipid-Liver | 1376 | 171 | -4.6764 | 177.7001 | 0.000E+00 | 0.000E+00 |
| | Average | Anthro-BP-Glucose | 1376 | 105 | -26.7622 | 105.0216 | 0.000E+00 | 0.000E+00 |
| | Lipid-Liver | Anthro-BP-Glucose | 171 | 105 | -22.3014 | 150.0289 | 0.000E+00 | 0.000E+00 |
| Total Cholesterol | Average | Lipid-Liver | 1376 | 171 | -7.9482 | 195.336 | 0.000E+00 | 0.000E+00 |
| | Average | Anthro-BP-Glucose | 1376 | 105 | -4.2568 | 111.1312 | 0.000E+00 | 1.000E-04 |
| | Lipid-Liver | Anthro-BP-Glucose | 171 | 105 | 1.0709 | 197.489 | 2.860E-01 | 8.580E-01 |
| Triglycerides | Average | Lipid-Liver | 1376 | 171 | -20.3273 | 174.601 | 0.000E+00 | 0.000E+00 |
| | Average | Anthro-BP-Glucose | 1376 | 105 | -6.7507 | 107.88 | 0.000E+00 | 0.000E+00 |
| | Lipid-Liver | Anthro-BP-Glucose | 171 | 105 | 11.1491 | 272.009 | 0.000E+00 | 0.000E+00 |
| Aspartate | Average | Lipid-Liver | 1376 | 171 | -3.521 | 175.1681 | 5.000E-04 | 1.600E-03 |
| | Average | Anthro-BP-Glucose | 1376 | 105 | 1.9415 | 118.8264 | 5.500E-02 | 1.650E-01 |
| | Lipid-Liver | Anthro-BP-Glucose | 171 | 105 | 4.0638 | 234.143 | 1.000E-04 | 2.000E-04 |
| Alanine | Average | Lipid-Liver | 1376 | 171 | -4.2566 | 176.3829 | 0.000E+00 | 1.000E-04 |
| | Average | Anthro-BP-Glucose | 1376 | 105 | -0.2754 | 118.8691 | 7.840E-01 | 1.000E+00 |
| | Lipid-Liver | Anthro-BP-Glucose | 171 | 105 | 3.683 | 244.7561 | 3.000E-04 | 9.000E-04 |
| Glutamyl | Average | Lipid-Liver | 1376 | 171 | -5.8033 | 171.8756 | 0.000E+00 | 0.000E+00 |
| | Average | Anthro-BP-Glucose | 1376 | 105 | -2.8266 | 108.7452 | 6.000E-03 | 1.800E-02 |
| | Lipid-Liver | Anthro-BP-Glucose | 171 | 105 | 3.9527 | 239.6927 | 1.000E-04 | 3.000E-04 |

**Supplementary Figure 1. Survival curve by cluster.**

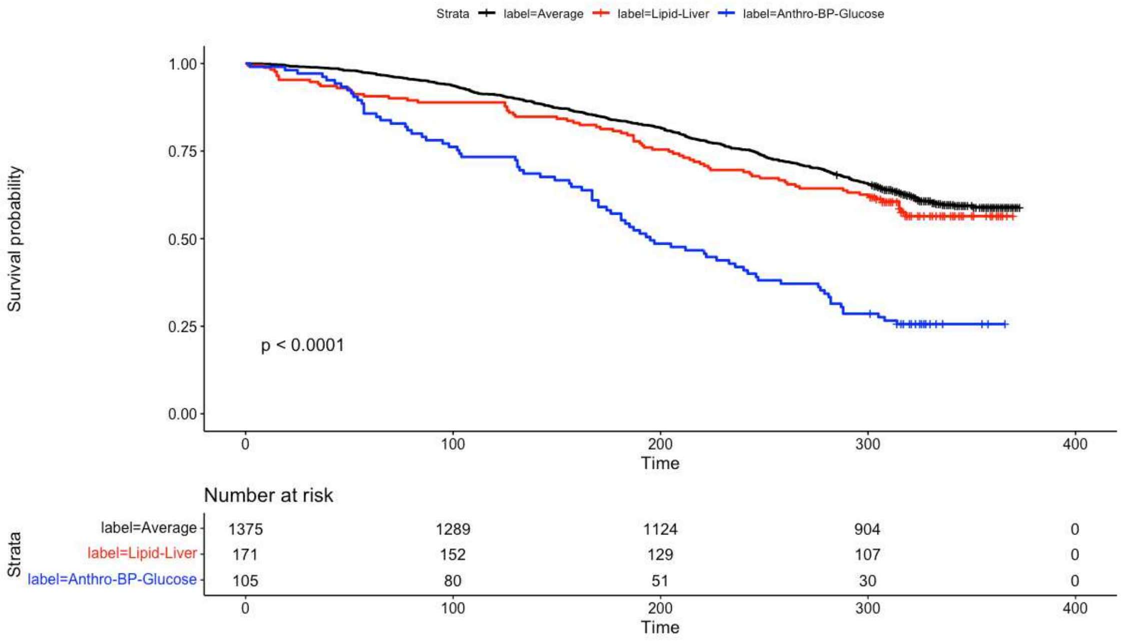




Carrillo-Larco RM, *et al. BMJ Open* 2022; 12:e067203. doi: 10.1136/bmjopen-2022-067203

**Supplementary Figure 2. Schoenfeld residuals for the crude Cox-proportional hazard model.**

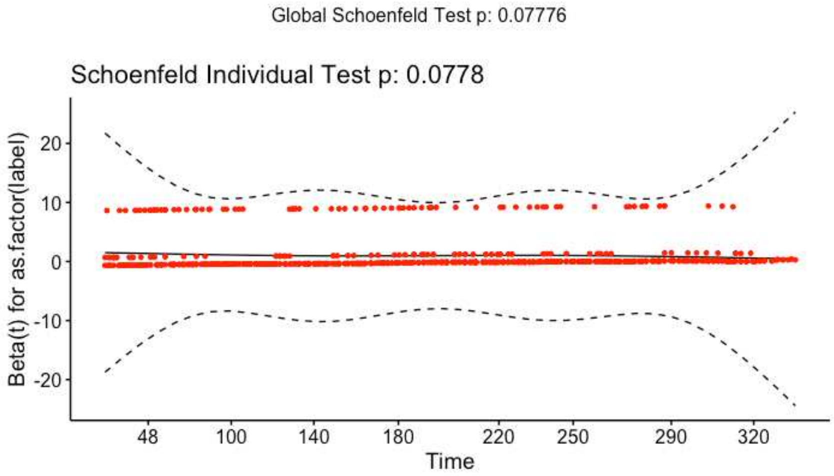




Carrillo-Larco RM, *et al. BMJ Open* 2022; 12:e067203. doi: 10.1136/bmjopen-2022-067203
