# Supplementary material for: Phenotypes of non-alcoholic fatty liver disease (NAFLD) and all-cause mortality: unsupervised machine learning analysis of NHANES III
Source: BMJ Open. 2022 Nov 23;12(11):e067203. doi: 10.1136/bmjopen-2022-067203 (PMC9685238; doi:10.1136/bmjopen-2022-067203)
Supplement: Author's manuscript [file bmjopen-2022-067203.draft_revisions.pdf]

BMJ Open is committed to open peer review. As part of this commitment we make the peer review history of every article we publish publicly available.

When an article is published we post the peer reviewers' comments and the authors' responses online. We also post the versions of the paper that were used during peer review. These are the versions that the peer review comments apply to.

The versions of the paper that follow are the versions that were submitted during the peer review process. They are not the versions of record or the final published versions. They should not be cited or distributed as the published version of this manuscript.

BMJ Open is an open access journal and the full, final, typeset and author-corrected version of record of the manuscript is available on our site with no access controls, subscription charges or pay-per-view fees (http://bmjopen.bmj.com).

If you have any questions on BMJ Open's open peer review process please email info.bmjopen@bmj.com

BMJ Open

Phenotypes of non-alcoholic fatty liver disease (NAFLD) and all-cause mortality: Unsupervised machine learning analysis of NHANES III

Journal:	*BMJ Open*
Manuscript ID	bmjopen-2022-067203
Article Type:	Original research
Date Submitted by the Author:	08-Aug-2022
Complete List of Authors:	Carrillo-Larco, Rodrigo; Imperial College London<br>Guzman-Vilca, Wilmer Cristobal; Universidad Peruana Cayetano Heredia; Universidad Peruana Cayetano Heredia,<br>Castillo-Cara, Manuel; Universidad Politécnica de Madrid, Artificial Intelligence Department<br>Alvizuri-Gómez, Claudia; Hospital Nacional Cayetano Heredia, Department of Gastroenterology<br>Alqahtani, Saleh; Johns Hopkins University, Department of Medicine<br>Garcia Larsen, Vanessa; Johns Hopkins University Bloomberg School of Public Health, Department of International Health
Keywords:	GASTROENTEROLOGY, EPIDEMIOLOGY, PREVENTIVE MEDICINE, PUBLIC HEALTH

SCHOLARONE™
Manuscripts

I, the Submitting Author has the right to grant and does grant on behalf of all authors of the Work (as defined in the below author licence), an exclusive licence and/or a non-exclusive licence for contributions from authors who are: i) UK Crown employees; ii) where BMJ has agreed a CC-BY licence shall apply, and/or iii) in accordance with the terms applicable for US Federal Government officers or employees acting as part of their official duties; on a worldwide, perpetual, irrevocable, royalty-free basis to BMJ Publishing Group Ltd ("BMJ") its licensees and where the relevant Journal is co-owned by BMJ to the co-owners of the Journal, to publish the Work in this journal and any other BMJ products and to exploit all rights, as set out in our licence.

The Submitting Author accepts and understands that any supply made under these terms is made by BMJ to the Submitting Author unless you are acting as an employee on behalf of your employer or a postgraduate student of an affiliated institution which is paying any applicable article publishing charge ("APC") for Open Access articles. Where the Submitting Author wishes to make the Work available on an Open Access basis (and intends to pay the relevant APC), the terms of reuse of such Open Access shall be governed by a Creative Commons licence – details of these licences and which Creative Commons licence will apply to this Work are set out in our licence referred to above.

Other than as permitted in any relevant BMJ Author's Self Archiving Policies, I confirm this Work has not been accepted for publication elsewhere, is not being considered for publication elsewhere and does not duplicate material already published. I confirm all authors consent to publication of this Work and authorise the granting of this licence.

For peer review only

Phenotypes of non-alcoholic fatty liver disease (NAFLD) and all-cause mortality: Unsupervised machine learning analysis of NHANES III

Rodrigo M Carrillo-Larco [1,2,3]

Wilmer Cristobal Guzman-Vilca [2,4,5]

Manuel Castillo-Cara [6]

Claudia Alvizuri-Gómez [7]

Saleh A Alqahtani [8,9]

Vanessa García-Larsen [10*]

1. Department of Epidemiology and Biostatistics, School of Public Health, Imperial College London, London, United Kingdom.
2. CRONICAS Centre of Excellence in Chronic Diseases, Universidad Peruana Cayetano Heredia, Lima, Peru.
3. Universidad Continental, Lima, Peru.
4. School of Medicine Alberto Hurtado, Universidad Peruana Cayetano Heredia, Lima, Peru.
5. Sociedad Científica de Estudiantes de Medicina Cayetano Heredia (SOCEMCH), Universidad Peruana Cayetano Heredia, Lima, Peru.
6. Ontology Engineering Group, Artificial Intelligence Department, Universidad Politécnica de Madrid, Madrid, Spain.
7. Department of Gastroenterology, Hospital Nacional Cayetano Heredia. Lima, Peru.
8. King Faisal Specialist Hospital & Research Centre, Riyadh 11564, Saudi Arabia.
9. Division of Gastroenterology and Hepatology, Department of Medicine, Johns Hopkins University, Baltimore, MD 21205, USA.
10. Program in Human Nutrition, Department of International Health, The Johns Hopkins Bloomberg School of Public Health, Baltimore, MD 21205, USA.

*Email: vgla@jhu.edu

For peer review only - http://bmjopen.bmj.com/site/about/guidelines.xhtml

ABSTRACT

Objectives: Non-alcoholic fatty liver disease (NAFLD) is a non-communicable disease with a rising prevalence worldwide and with large burden for patients and health systems. To date, the presence of unique phenotypes in NAFLD patients has not been studied, and their identification could inform precision medicine and public health with pragmatic implications in personalized management and care for NAFLD patients.

Design: Cross-sectional and prospective (up to December 31st, 2019) analysis of NHANES III (1988-1994).

Primary and secondary outcomes measures: NAFLD diagnosis was based on liver ultrasound. The following predictors informed an unsupervised machine learning algorithm (k-means): body mass index; waist circumference; systolic blood pressure (SBP); plasma glucose; total cholesterol; triglycerides; liver enzymes alanine aminotransferase, aspartate aminotransferase, and gamma glutamyl transferase. We summarized (means) and compared the predictors across clusters. We used Cox Proportional Hazard models to quantify the all-cause mortality risk associated with each cluster.

Results: 1,652 patients with NAFLD (mean age 47.2 years and 51.5% women) were grouped into three clusters: Anthro-SBP-Glucose (6.36%; highest levels of anthropometrics, SBP and glucose); Lipid-Liver (10.35%; highest levels of lipid and liver enzymes); and Average (83.29%; predictors at average levels). Compared to the Average phenotype, the Anthro-SBP-Glucose phenotype had higher all-cause mortality risk (aHR=2.88; 95% CI: 2.26-3.67); the Lipid-Liver phenotype was not associated with higher all-cause mortality risk (aHR=1.11; 95% CI: 0.86-1.42).

Conclusions: There is heterogeneity in NAFLD patients, whom can be divided into three phenotypes with different mortality risk. These phenotypes could guide specific interventions and management plans, thus advancing precision medicine and public health for NAFLD patients.

Keywords: data-driven; artificial intelligence; non-communicable diseases; precision medicine.

For peer review only - http://bmjopen.bmj.com/site/about/guidelines.xhtml

Strengths and limitations of this study

- We leveraged on population-based data with image-based NAFLD diagnosis and long-term follow-up.
- We applied machine learning techniques to identify phenotypes amongst people with NAFLD, consistent with precision medicine and precision public health.
- Arguably, the gold standard for NAFLD diagnosis is liver biopsy, though given the source of the dataset (population-based nationally representative study), taking biopsies was not possible.
- We did not use diet-related variables because measuring these may vary between places and observers unlike blood biomarkers.

For peer review only

For peer review only - http://bmjopen.bmj.com/site/about/guidelines.xhtml

INTRODUCTION

The epidemiology of non-communicable diseases (NCDs) is largely driven by cardiometabolic risk factors and diseases, namely dyslipidaemias, type 2 diabetes mellitus (T2DM), hypertension, and cardiovascular diseases. Nonetheless, there are other NCDs rapidly growing along with, and as a consequence of,[1,2] the aforementioned cardiometabolic conditions. Non-alcoholic fatty liver disease (NAFLD) is an outstanding example with a soaring prevalence worldwide,[3-6] high economic costs for patients and health systems,[7,8] and poor patient-oriented outcomes.[9] Despite this high disease burden, there are no specific treatments for NAFLD other than managing the underlying conditions like obesity or T2DM together with recommendations for healthy lifestyles.[10-14] Consistent with the global call for precision medicine and public health, a way to maximize the benefits of available and forthcoming[15-17] treatments for NAFLD, could be to identify phenotypes in NAFLD patients. Therefore, specific management plans can be proposed according to the underlying profile of each phenotype. A data-driven machine learning (ML) approach has been proven to be a reliable method to identify phenotypes amongst patients, and there are several examples for T2DM.[18] However, NAFLD research has not yet used ML to identify phenotypes or subpopulations, even though it could signal groups of patients for which different management plans can be provided and different prognosis can be expected. We aimed to identify phenotypes in people with NAFLD in the general population and to quantify the all-cause mortality risk associated with each phenotype.

METHODS

Study design and data sources

This is a data-driven analysis following an unsupervised ML approach. We conducted a cross-sectional analysis whereby we identified clusters of NAFLD patients and described the underling phenotype of each cluster. We also conducted a prospective analysis whereby we investigated whether the phenotypes were associated with higher all-cause mortality risk.

We used individual-level data from the National Health and Nutrition Examination Survey (NHANES) III conducted between 1988-1994. The NHANES III included a nationally representative sample of non-institutionalized individuals in the United States of America (USA).[19]

For peer review only - http://bmjopen.bmj.com/site/about/guidelines.xhtml

Study population

The study population included people aged between 20 and 74 years who had hepatic imaging data. Hepatic imaging refers to ultrasound examinations conducted following standard procedures to secure consistent and reliable results for all participants.[20] We included people whose imaging results were deemed "confident" or "absolute" to secure high-quality data for the definition of NAFLD.[20] For the analyses, we only included people with NAFLD defined as a "moderate-severe" hepatic steatosis.[20]

People with a positive test for Hepatitis B (HBsAG) and Hepatitis C (antiHCV) were excluded, and so were people with high alcohol consumption (Supplementary Materials p. 02). People whose risk factors levels were below or above these plausibility thresholds were excluded too: body mass index (BMI) between 10 kg/m^2 and 80 kg/m^2; waist circumference between 30 cm and 200 cm; systolic blood pressure (SBP) between 70 mmHg and 270 mmHg; plasma glucose between 45 mg/dL and 540 mg/dL; total cholesterol between 20 mg/dL and 773 mg/dL; and triglycerides between 17 mg/dL and 1,771 mg/dL. Details about data preparation and selection of the study population are available in Supplementary Materials p. 02.

Variables

From the NHANES dataset we selected ten predictors, which will inform the composition of the clusters and characterize the phenotype of each cluster. These predictors were selected because they are established cardiometabolic risk factors, and cardiovascular diseases are a leading cause of mortality in people with NAFLD.[21] We also included liver biomarkers as predictors because NAFLD is a chronic liver condition. The predictors included chronological age (years); BMI (kg/m^2) based on measured weight and height; waist circumference (cm); SBP (mmHg); plasma glucose (mg/dL) which was used regardless of fasting duration (41.53% or 686/1,652 had a fasting duration <8 hours) to maximize sample size for the study population comprising only people with NAFLD; total cholesterol (mg/dL); triglycerides (mg/dL); serum alanine aminotransferase (U/L); serum aspartate aminotransferase (U/L); and serum gamma glutamyl transferase (U/L; GGT). All blood biomarkers where collected and analysed following standard procedures.[22] We conducted a completed-case analysis for the ten predictors of interest.

For peer review only - http://bmjopen.bmj.com/site/about/guidelines.xhtml

We did not include more predictors, such as coagulation or anaemia biomarkers, to deliver a model that can be replicated in many places and by researchers who may not have access to more sophisticated biomarkers. Including more or more sophisticated predictors would limit the use of our model to places where these predictors are available. This would, most likely, exclude several primary healthcare facilities and those in rural or resource-limited settings.

Mortality data are also available for NHANES III.[23] The National Centre for Health Statistics (NCHS) delivers a mortality dataset linked to NHANES participants (adults only). Follow-up duration, in person months, is computed from the examination date to the date of death or censorship (December 31st, 2019). Because a priori we did not know the number of clusters or their underlying profiles, we focused on all-cause mortality rather than on a specific cause of death.

For peer review only

Analysis

Open-access resources

The analyses were conducted in Python 3.10 using PyCharm 2021.3.3. and Jupyter Notebooks; we also used R 4.1.2 for the statistical analyses. The Jupyter Notebooks and R scripts are available as Supplementary Materials. All analytical methods are detailed in Supplementary Materials pp. 02-05.

Unsupervised machine learning analysis

We applied principal components analysis (PCA) to the ten predictors. We chose four principal components because these explained 95.19% of the variance. In other words, we retained 95.19% of the information in the original dataset with these four components. On these four principal components, we applied the k-means algorithm, an unsupervised ML method, to identify clusters of NAFLD patients with unique phenotypes. To define the number of clusters, which is unknown at the beginning of any unsupervised ML analysis, we followed a data-driven approach together with expert knowledge about NAFLD. First, we plotted a dendrogram with Euclidean distances, which suggested to use five clusters. Second, the Elbow method suggested to use five or six clusters. Third, the Silhouette score was highest with two clusters,

For peer review only - http://bmjopen.bmj.com/site/about/guidelines.xhtml

followed by three clusters. Fourth, the Jaccard score was highest for three clusters. Therefore, based on the above metrics and expert knowledge, we decided on three clusters. In exploratory analyses we also tried selecting the number of clusters based on other algorithms and the results were consistent with those herein summarized; the other algorithms we tried were: Balanced Iterative Reducing and Clustering using Hierarchies (BIRCH); spectral; and agglomerative.

Statistical analysis

First, we conducted a descriptive analysis to characterize the three clusters in terms of the ten predictors. That is, we summarized the mean of the ten predictors in each cluster and used Boxplots. We used t-tests for pair-wise comparisons between all clusters; the p-value is reported accounting for the Bonferroni correction for multiple comparisons.

Second, to study the all-cause mortality associated with each cluster, we ran Cox Proportional Hazard models reporting Hazard Ratios (HR) along with the 95% Confidence Interval (95% CI). We used time in the study as the time scale in the Cox models. We fitted three models: i) crude model only including the outcome (all-cause mortality) and the exposure (clusters, categorical variable with three levels); ii) model 1, including sex; and iii) model 2, including sex, smoking status (current smoker of cigarettes, no vs yes), years of education, and household income (<20,000$ vs ≥20,000$ in the last year). The proportional hazard assumption was verified in the crude model, and we did not observe evidence that this assumption was violated. We did not adjust for age or other cardio-metabolic risk factors because these were included in the clusters which are the main predictors (i.e., independent variable) in the regression models.

The statistical analysis did not account for the complex survey design of the NHANES survey (e.g., sampling weights). We aimed to characterize the clusters and study if they were associated with higher all-cause mortality risk, rather than to provide prevalence estimates or means what are representative at the national level.

For peer review only - http://bmjopen.bmj.com/site/about/guidelines.xhtml

Sensitivity analysis

We carried several sensitivity analyses to elucidate if any observed differences were driven by the fact that we did not only include people with more than eight hours of fasting. First, we compared the mean plasma glucose levels across phenotypes restricting the sample to those with eight or more hours of fasting duration. Second, we ran the fully adjusted Cox model (model 2) restricting the sample to those with eight or more hours of fasting. Third, we ran the fully adjusted Cox model (model 2) including fasting duration (in hours) as an additional confounder rather than restricting the sample to those with eight or more hours of fasting duration. To explore whether the phenotypes were associated with all-cause mortality above and beyond the individual risk factors, we also ran model 2 including the 10 predictors as confounders.

Role of the funding source

This study received no funding. The opinions on this document are those from the authors alone and do not represent the views of the institutions to which they belong. The first two authors collated, pooled, and analysed the data; the first two authors vouch for the accuracy of the results.

Patient and public involvement

It was not appropriate or possible to involve patients or the public in the design, or conduct, or reporting, or dissemination plans of our research.

Ethics

We analyzed open access data at the country level. Human subjects were not directly involved in this work. This work was deemed as of minimal risk.

RESULTS

Study population

There were 1,652 people with NAFLD, the mean age was 47.2 years and 51.5% were women. Across the study population the mean BMI was 30.0 kg/m^2 and the mean waist circumference was 100.7 cm; the mean

For peer review only - http://bmjopen.bmj.com/site/about/guidelines.xhtml
For peer review only

SBP was 128.9 mmHg, the mean plasma glucose was 112.9 mg/dL, the mean total cholesterol was 211.3 mg/dL, and the mean triglycerides was 191.8 mg/dL; the mean aspartate was 24.5 U/L, the mean alanine was 23.5 U/L and the mean GGT was 40.7 U/L.

Cluster profiles

Given the underlying profiles of each phenotype (Table 1 and Figure 1), these were labelled as it follows: Lipid-Liver (i.e., highest levels of cholesterol and liver enzymes); Anthro-SBP-Glucose (i.e., highest levels of anthropometrics, SBP and glucose); and Average (i.e., mean levels of the predictors were always in between the former two phenotypes). The Average phenotype grouped 1,376 people (83.29%), followed by the Lipid-Liver (171 people, 10.35%), and the Anthro-SBP-Glucose phenotype with 105 people (6.36%).

For peer review only

For peer review only - http://bmjopen.bmj.com/site/about/guidelines.xhtml

Table 1. General characteristics of participants by phenotype cluster

Clusters	Age	BMI	Waist	SBP	Glucose	TC	Triglycerides	Aspartate	Alanine	GGT
Average	46.6	29.9	100.0	127.9	99.3	205.9	149.7	23.6	21.9	33.7
Lipid-Liver	47.1	30.2	103.3	132.5	114.9	241.0	495.7	34.6	36.5	93.0
Anthro-SBP-Glucose	55.3	31.7	106.1	135.9	288.4	232.9	248.0	20.7	22.4	48.0

All variables are expressed as means; the standard deviations for each of these variables are shown in Supplementary Table 1. Comparisons across clusters are shown in Figure 1 and Supplementary Table 2. BMI: body mass index (kg/m^2); waist refers to waist circumference (cm); SBP: systolic blood pressure (mmHg); glucose refers to plasma glucose (mg/dL); TC: total cholesterol (mg/dL); triglycerides are expressed in mg/dL; aspartate refers to aspartate aminotransferase (AST; U/L); alanine refers to alanine aminotransferase (ALT; U/L); GGT: Gamma-glutamyl transferase (U/L)

For peer review only

Differences between the Average and the Lipid-Liver phenotypes were always statistically significant, except for age (p=1) and BMI (p=1) (Figure 1 and Supplementary Table 2). Differences between the Average and Anthro-SBP-Glucose phenotypes were always statistically significant except for alanine (p=1) and aspartate (p=0.165). Differences between the Lipid-Liver phenotype and the Anthro-SBP-Glucose phenotype were significant for six predictors: age (older in the Anthro-SBP-Glucose); plasma glucose (higher in the Anthro-SBP-Glucose); triglycerides (higher in the Lipid-Liver); alanine (higher in the Lipid-Liver); aspartate (higher in the Lipid-Liver); and GGT (higher in the Lipid-Liver). That is, most of the unique features of the Lipid-Liver phenotype were different –higher– in comparison to the Anthro-SBP-Glucose phenotype; conversely, only glucose in the Anthro-SBP-Glucose phenotype was significantly different –higher– relative to the Lipid-Liver phenotype.

All-cause mortality

Amongst those who died, 78.0% were in the Average phenotype; 10.5% were in the Anthro-SBP-Glucose phenotype, and 11.5% were in the Lipid-Liver phenotype. The mean follow-up duration was 270.1 months (~22.5 years), ranging from a month to 373 months. The survival probability decreased much faster for the Anthro-SBP-Glucose phenotype than for the Lipid-Liver phenotype (Supplementary Figure 1).

The crude Cox proportional hazard model showed that, in comparison to the Average phenotype, the Lipid-Liver was not associated with higher all-cause mortality (HR=1.17; p=0.217); conversely, the Anthro-SBP-Glucose phenotype were associated with higher all-cause mortality risk (HR=2.94; p<0.001; Table 2). After adjusting for sex, the association for the Lipid-Liver phenotype was still not significant (HR=1.11; p=0.431), and the association for the Anthro-SBP-Glucose phenotype remained significant (HR=2.97; p<0.001). The same pattern was observed when we further adjusted for smoking, education, and household income whereby the Lipid-Liver phenotype was not associated with higher all-cause mortality risk (HR=1.21; p=0.427), yet the Anthro-SBP-Glucose phenotype was still strongly associated with higher all-cause mortality risk (HR=2.88; p<0.001).

For peer review only - http://bmjopen.bmj.com/site/about/guidelines.xhtml

Table 2. Mortality risk in NAFLD patients by phenotype cluster

Cluster	Outcome: all-cause mortality (HR, 95% CI)*		
	**Crude** N=1,651 Events = 678	**Model 1** N=1,651 Events = 678	**Model 2** N=1,622 Events = 663
Average	1	1	1
Lipid-Liver	1.17 (0.91-1.50)	1.11 (0.86-1.42)	1.11 (0.86-1.42)
Anthro-SBP-Glucose	2.94 (2.32-3.74)	2.97 (2.34-3.78)	2.88 (2.26-3.67)

*Cox proportional hazard ratios; Model 1 included sex. Model 2 included, in addition to sex, smoking status (current, no vs yes); years of education (numeric variable); and household income (<20,000$ vs ≥20,000$ in the last year). For the crude model, the global test for the proportional-hazard assumption was not significant (p=0.078), suggesting this assumption holds which is also supported by the Schoenfeld residuals plot (Supplementary Figure 2).

For peer review only

Sensitivity analyses

The Anthro-SBP-Glucose phenotype still had the highest mean plasma glucose (273.5 mg/dL), followed by the Lipid-Liver phenotype (115.9 mg/dL), and the Average phenotype (101.2 mg/dL). The p-value for the Bonferroni-adjusted t-test for all pairwise comparisons was significant (p<0.003).

When model 2 for the Cox regression was restricted to people with eight or more hours of fasting, the results were consistent with the main analysis: the Anthro-SBP-Glucose phenotype was strongly associated with higher all-cause mortality risk (HR=2.65; 95% CI: 1.90-3.70; p<0.001), and the association for the Lipid-Liver phenotype was not significant (HR=1.12; 95% CI: 0.80-1.58; p=0.512). Virtually the same results were observed when model 2 included fasting duration as a covariate rather than restricting the sample to those with eight or more hours of fasting duration: HR=2.89 (95% CI: 2.27-3.69; p<0.001) for the Anthro-SBP-Glucose, and HR=1.11 (95% CI: 0.86-1.43; p=0.414) for the Lipid-Liver phenotype.

When model 2 also included the 10 predictors used to define the phenotypes, neither the Lipid-Liver (HR=1.20; 95% CI: 0.82-1.74; p=0.342) phenotype nor the Anthro-SBP-Glucose (HR=1.46; 95% CI: 0.90-2.38; p=0.126) phenotype was associated with higher all-cause mortality risk.

DISCUSSION

Main results

In a sample of NAFLD patients from the general population and using simple predictors, a ML analysis revealed three phenotypes with unique profiles. The Anthro-SBP-Glucose phenotype had the highest mean levels of BMI, waist circumference, SBP and plasma glucose. Patients in the Lipid-Liver phenotype had, on average, the highest levels of cholesterol and liver biomarkers. There was a third phenotype whose members had average risk factor levels. Most NAFLD patients belonged to the Average phenotype (83%), followed by the Lipid-Liver (10%) and Anthro-SBP-Glucose (6%) phenotypes. In comparison to the Average phenotype, the Anthro-SBP-Glucose phenotype had higher all-cause mortality risk; the all-cause mortality risk appeared not to be different between the Average and Lipid-Liver phenotypes.

For peer review only - http://bmjopen.bmj.com/site/about/guidelines.xhtml

We believe that these findings have the potential to advance precision medicine and public health by identifying variations in the clinical and metabolic presentation of NAFLD. Such variations suggest three homogenous phenotypes. NAFLD patients with the Anthro-SBP-Glucose phenotype, which was associated with the highest all-cause mortality risk, should receive urgent counselling and medication to reduce weight, SBP and glucose levels. While maintaining a healthy lifestyle should always be a cornerstone in the management of NAFLD patients, those with the Lipid-Liver phenotype could most benefit from novel therapies targeting cholesterol and liver physiology.[15-17] The Average phenotype was the most frequent. This signals a group of NAFLD patients who should be carefully monitored, though, perhaps, not with the same frequency or intensity as for the other phenotypes in which risk factors levels were higher and had higher all-cause mortality risk.

Results in context

NAFLD, defined as >5% of hepatic steatosis in images or histology in the absence of secondary causes (e.g., viral hepatitis),[11] is the most common cause of chronic liver disease in the last decade.[24] Although NAFLD is distributed worldwide, the prevalence of NAFLD is particularly high in the Middle East and South America.[24] Future research in these world regions could elaborate on our work to trial specific recommendations according to the patient's cluster.

To date, there are no specific treatments to prevent or cure NAFLD. Vitamin E has been proposed as a potential therapy for NAFLD, because it may improve non-alcoholic steatohepatitis. However, systematic reviews and meta-analysis of randomised trials have shown inconsistent evidence to recommend vitamin E for NAFLD;[25] moreover, potential adverse effects of vitamin E should be taken into consideration.[26] Current strategies focus on weight loss, exercise and diet. Mediterranean diet appears to reduce hepatic steatosis due to reduction in insulin-resistance and total lipid concentrations;[27] likewise, weight loss of ≥7% correlates with improvement of histological features.[26] Overall, implementing these interventions to improve healthy lifestyles should be the standard of care for patients with NAFLD, and more so for those in the Anthro-SBP-Glucose phenotype herein found.

For peer review only - http://bmjopen.bmj.com/site/about/guidelines.xhtml
For peer review only

In 2020, an international consensus proposed the term *metabolic associated fatty liver disease* (MAFLD) to substitute NAFLD due to the strong metabolic component of this chronic liver condition.[28] Our work may support the MAFLD nomenclature because the Anthro-SBP-Glucose phenotype, largely consisting of traits consequence of metabolic dysfunction, had the highest all-cause mortality risk. Nevertheless, our work also signals there is heterogeneity in patients with NAFLD which may better guide treatment options above and beyond one specific nomenclature (NAFLD vs MAFLD).

Few studies have quantified the association between phenotypes and mortality in patients with NAFLD. Golabi *et al*,[29] evaluated the effect of abdominal and overall adiposity on the mortality risk in patients with NAFLD; after 22.4 years of follow-up, they observed that patients with NAFLD and normal BMI but high waist circumference, had higher risk of cardiovascular mortality.[29] This is consistent with the Anthro-SBP-Glucose phenotype having the highest all-cause mortality risk.

Strengths and limitations

We leveraged on population-based data with image-based[20] NAFLD diagnosis and long-term follow-up.[23] We applied ML techniques to identify phenotypes amongst people with NAFLD, consistent with precision medicine and precision public health. Not only did we describe the underlying profile of each phenotype, but we also quantified the all-cause mortality risk associated with each phenotype.

Notwithstanding, there are limitations we should acknowledge. First, the gold standard to diagnose NAFLD requires a liver biopsy. Collecting a liver biopsy from a large population-based sample as that of NHANES would be (almost) impossible. From a clinical perspective, the fact that NAFLD was diagnosed with liver ultrasound could be a limitation; however, from a global or population health perspective, it could be a step forward to characterize phenotypes in people with NAFLD. Second, aiming to deliver a friendly and easy to replicate model, we included ten predictors that may be accessible to many clinicians and researchers. Had we used more sophisticated predictors, it would limit the reproducibility of our model by a broad audience. However, this decision could have led to the omission of other potential phenotypes. For example, if we had included anaemia biomarkers (e.g., haemoglobin or ferritin), there could have been an additional

For peer review only - http://bmjopen.bmj.com/site/about/guidelines.xhtml

For peer review only

phenotype characterized by anaemia. Our work opens the door to use ML in NAFLD research aiming for precision medicine and public health, and future work could expand our set of phenotypes for specific setting where access to these predictors is not a limitation. In this line, although diet is a key variable in NAFLD, we did not include diet-based variables in the cluster analysis; this, because, while inexpensive, collecting diet information may be troublesome, may require of specific tools (e.g., 24-hour diet recalls), and may need context-expert knowledge to ask about foods consumed locally. As argued before for more sophisticated biomarkers, including diet profiles could also limit the applicability of the clusters.

Conclusions

There is heterogeneity in NAFLD patients, whom can be divided into three phenotypes. These phenotypes are either characterized by high levels of anthropometrics, SBP and glucose, or by high levels of cholesterol and liver biomarkers. These phenotypes could guide specific interventions, together with personalized management and follow-up plans thus advancing precision medicine and public health for NAFLD patients.

For peer review only

For peer review only - http://bmjopen.bmj.com/site/about/guidelines.xhtml

Contributions

RMC-L and VG-L conceived the research question. RMC-L pooled and processed the datasets with support from WCG-V. RMC-L conducted the machine learning analysis with support from MC-C. RMC-L conducted the statistical analysis. RMC-L drafted the first version of the manuscript with support from all co-authors. All authors provided critical input, commented, and edited the manuscript. All authors approved the submitted version.

Data availability

All NHANES datasets are publically available and can be download from the official website: https://www.cdc.gov/nchs/nhanes/index.htm All analysis code (Jupyter Notebooks and R Scripts) are available as Supplementary Materials.

Funding

RMC-L is supported by a Wellcome Trust International Training Fellowship (Wellcome Trust 214185/Z/18/Z).

Conflict of interests

None to declare.

Patients consent

Not applicable

Permission to reproduce materials from other sources

Not applicable

Clinical trial registration

Not applicable

For peer review only

FIGURES

Figure 1. Distribution of the predictors by phenotype cluster

P-values are for pair-wise comparisons and Bonferroni-adjusted for multiple comparisons (Supplementary Table 1). BMI: body mass index (kg/m^2); GGT: Gamma-glutamyl transferase (U/L).

For peer review only

Ethics

We analyzed open access data at the country level. Human subjects were not directly involved in this work.

This work was deemed as of minimal risk.

For peer review only

For peer review only - http://bmjopen.bmj.com/site/about/guidelines.xhtml

REFERENCES

1. Stefan N, Cusi K. A global view of the interplay between non-alcoholic fatty liver disease and diabetes. *The lancet Diabetes & endocrinology* 2022; **10**(4): 284-96.

2. Sarwar R, Pierce N, Koppe S. Obesity and nonalcoholic fatty liver disease: current perspectives. *Diabetes, metabolic syndrome and obesity : targets and therapy* 2018; **11**: 533-42.

3. Le MH, Yeo YH, Li X, et al. 2019 Global NAFLD Prevalence: A Systematic Review and Meta-analysis. *Clinical gastroenterology and hepatology : the official clinical practice journal of the American Gastroenterological Association* 2021.

4. Li J, Zou B, Yeo YH, et al. Prevalence, incidence, and outcome of non-alcoholic fatty liver disease in Asia, 1999-2019: a systematic review and meta-analysis. *The lancet Gastroenterology & hepatology* 2019; **4**(5): 389-98.

5. Rojas YAO, Cuellar CLV, Barrón KMA, Verdugo JPA, Miranda AL. Non-alcoholic Fatty Liver Disease prevalence in Latin America: A systematic review and meta-analysis. *Annals of hepatology* 2022: 100706.

6. Spearman CW, Afihene M, Betiku O, et al. Epidemiology, risk factors, social determinants of health, and current management for non-alcoholic fatty liver disease in sub-Saharan Africa. *The lancet Gastroenterology & hepatology* 2021; **6**(12): 1036-46.

7. Younossi ZM, Blissett D, Blissett R, et al. The economic and clinical burden of nonalcoholic fatty liver disease in the United States and Europe. *Hepatology (Baltimore, Md)* 2016; **64**(5): 1577-86.

8. Hagström H, Nasr P, Ekstedt M, et al. Health Care Costs of Patients With Biopsy-Confirmed Nonalcoholic Fatty Liver Disease Are Nearly Twice Those of Matched Controls. *Clinical gastroenterology and hepatology : the official clinical practice journal of the American Gastroenterological Association* 2020; **18**(7): 1592-9.e8.

9. Younossi ZM, Henry L. Economic and Quality-of-Life Implications of Non-Alcoholic Fatty Liver Disease. *PharmacoEconomics* 2015; **33**(12): 1245-53.

10. LaBrecque DR, Abbas Z, Anania F, et al. World Gastroenterology Organisation global guidelines: Nonalcoholic fatty liver disease and nonalcoholic steatohepatitis. *Journal of clinical gastroenterology* 2014; **48**(6): 467-73.

For peer review only - http://bmjopen.bmj.com/site/about/guidelines.xhtml

11. Chalasani N, Younossi Z, Lavine JE, et al. The diagnosis and management of nonalcoholic fatty liver disease: Practice guidance from the American Association for the Study of Liver Diseases. *Hepatology (Baltimore, Md)* 2018; **67**(1): 328-57.

12. Leoni S, Tovoli F, Napoli L, Serio I, Ferri S, Bolondi L. Current guidelines for the management of non-alcoholic fatty liver disease: A systematic review with comparative analysis. *World journal of gastroenterology* 2018; **24**(30): 3361-73.

13. Arab JP, Dirchwolf M, Álvares-da-Silva MR, et al. Latin American Association for the study of the liver (ALEH) practice guidance for the diagnosis and treatment of non-alcoholic fatty liver disease. *Annals of hepatology* 2020; **19**(6): 674-90.

14. European Association for the Study of the Liver (EASL), European Association for the Study of Diabetes (EASD), European Association for the Study of Obesity (EASO). EASL-EASD-EASO Clinical Practice Guidelines for the management of non-alcoholic fatty liver disease. *Diabetologia* 2016; **59**(6): 1121-40.

15. Mantovani A, Byrne CD, Targher G. Efficacy of peroxisome proliferator-activated receptor agonists, glucagon-like peptide-1 receptor agonists, or sodium-glucose cotransporter-2 inhibitors for treatment of non-alcoholic fatty liver disease: a systematic review. *The lancet Gastroenterology & hepatology* 2022; **7**(4): 367-78.

16. Ferguson D, Finck BN. Emerging therapeutic approaches for the treatment of NAFLD and type 2 diabetes mellitus. *Nature reviews Endocrinology* 2021; **17**(8): 484-95.

17. Fatima K, Moeed A, Waqar E, et al. Efficacy of statins in treatment and development of non-alcoholic fatty liver disease and steatohepatitis: A systematic review and meta-analysis. *Clinics and research in hepatology and gastroenterology* 2021; **46**(4): 101816.

18. Sarría-Santamera A, Orazumbekova B, Maulenkul T, Gaipov A, Atageldiyeva K. The Identification of Diabetes Mellitus Subtypes Applying Cluster Analysis Techniques: A Systematic Review. *International journal of environmental research and public health* 2020; **17**(24).

19. Centers for Disease Control and Prevencion. National Center for Health Statistics. National Health and Nutrition Examination Survey. NHANES III (1988-1994). Available at: https://wwwn.cdc.gov/nchs/nhanes/nhanes3/default.aspx (Accessed May 18th, 2022).

For peer review only - http://bmjopen.bmj.com/site/about/guidelines.xhtml

20. National Health and Nutrition Examination Survey. 1988-1994 Data Documentation, Codebook, and Frequencies. Hepatic/Gallbladder Ultrasound and Hepatic Steatosis (HGUHS). Available at: https://wwwn.cdc.gov/nchs/data/nhanes3/34a/HGUHS.htm (Accessed May 18th, 2022).

21. Mahfood Haddad T, Hamdeh S, Kanmanthareddy A, Alla VM. Nonalcoholic fatty liver disease and the risk of clinical cardiovascular events: A systematic review and meta-analysis. *Diabetes & metabolic syndrome* 2017; **11 Suppl 1**: S209-s16.

22. Centers for Disease Control and Prevention. NHANES III - Laboratory Data File. Available at: https://wonder.cdc.gov/wonder/sci_data/surveys/hanes/hanes3/type_txt/lab.asp (Accessed May 18th 2022).

23. National Center for Health Statistics. 2019 Public - Use Linked Mortality Files. Available at: https://www.cdc.gov/nchs/data-linkage/mortality-public.htm (Accessed 19th May 2022).

24. Younossi Z, Anstee QM, Marietti M, et al. Global burden of NAFLD and NASH: trends, predictions, risk factors and prevention. *Nature reviews Gastroenterology & hepatology* 2018; **15**(1): 11-20.

25. Amanullah I, Khan YH, Anwar I, Gulzar A, Mallhi TH, Raja AA. Effect of vitamin E in non-alcoholic fatty liver disease: a systematic review and meta-analysis of randomised controlled trials. *Postgraduate medical journal* 2019; **95**(1129): 601-11.

26. European Association for the Study of the Liver (EASL), European Association for the Study of Diabetes (EASD), (EASO) EAftSoO. EASL-EASD-EASO Clinical Practice Guidelines for the management of non-alcoholic fatty liver disease. *Diabetologia* 2016; **59**(6): 1121-40.

27. Plaz Torres MC, Aghemo A, Lleo A, et al. Mediterranean Diet and NAFLD: What We Know and Questions That Still Need to Be Answered. *Nutrients* 2019; **11**(12).

28. Eslam M, Sanyal AJ, George J. MAFLD: A Consensus-Driven Proposed Nomenclature for Metabolic Associated Fatty Liver Disease. *Gastroenterology* 2020; **158**(7): 1999-2014.e1.

29. Golabi P, Paik JM, Arshad T, Younossi Y, Mishra A, Younossi ZM. Mortality of NAFLD According to the Body Composition and Presence of Metabolic Abnormalities. *Hepatology communications* 2020; **4**(8): 1136-48.

For peer review only

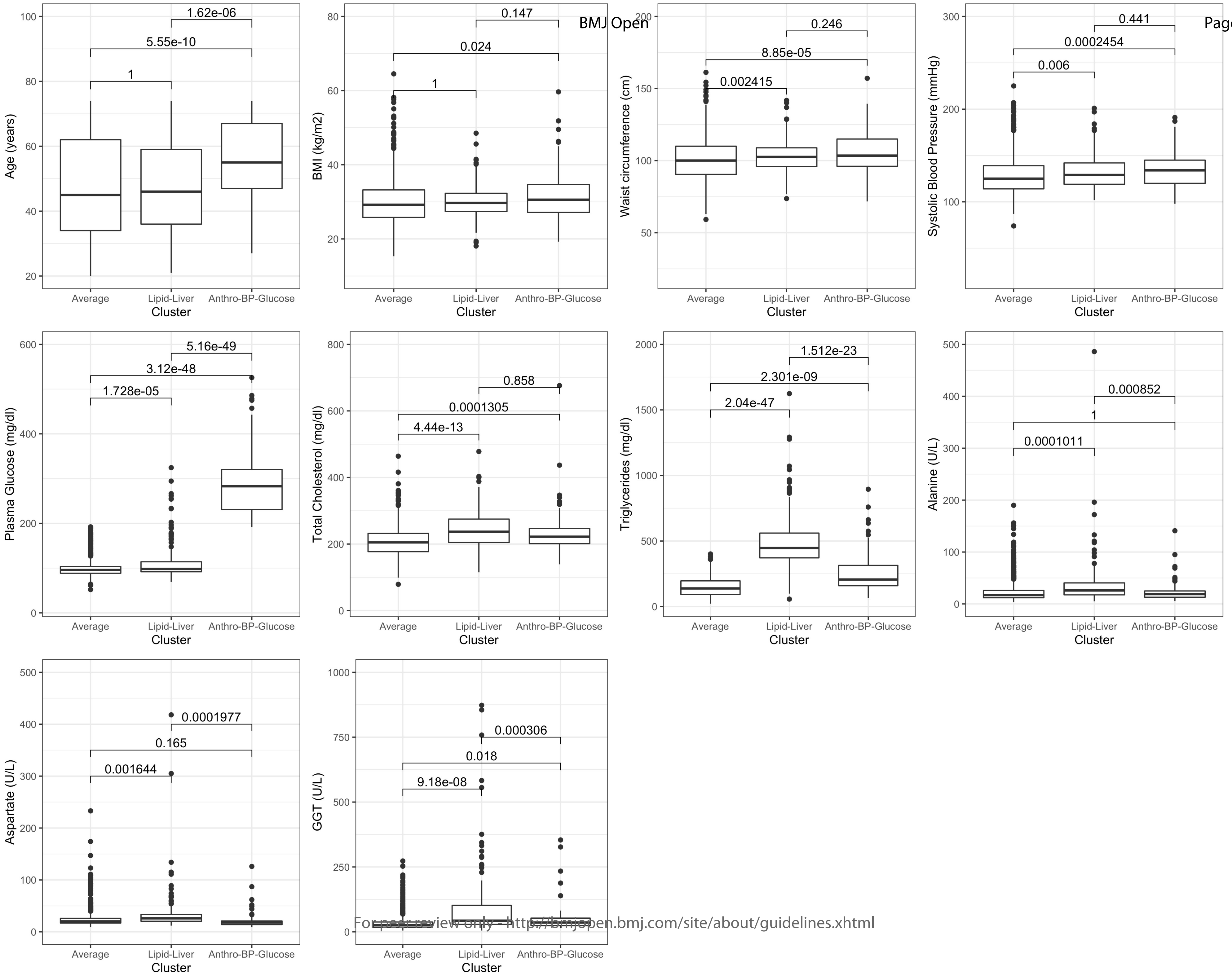
Age (years)
BMI (kg/m2)
Waist circumference (cm)
Systolic Blood Pressure (mmHg)
Plasma Glucose (mg/dl)
Total Cholesterol (mg/dl)
Triglycerides (mg/dl)
Alanine (U/L)
Aspartate (U/L)
GGT (U/L)
Cluster
Average
Lipid-Liver
Anthro-BP-Glucose
1.62e-06
5.55e-10
1
0.147
0.024
1
0.246
8.85e-05
0.002415
0.441
0.0002454
0.006
5.16e-49
3.12e-48
1.728e-05
0.858
0.0001305
4.44e-13
1.512e-23
2.301e-09
2.04e-47
0.000852
1
0.0001011
0.0001977
0.165
0.001644
0.000306
0.018
9.18e-08

SUPPLEMENTARY MATERIALS

Phenotypes of non-alcoholic fatty liver disease (NAFLD) and all-cause mortality: Unsupervised machine learning analysis with NHANES III

Table of Contents

Supplementary Methods ... 2

Data preparation ... 2

Study sample ... 2

Number of clusters ... 3

Supplementary Table 1. Underlying characteristics of each cluster. ... 7

Supplementary Table 2. Pair-wise comparisons between clusters. ... 8

Supplementary Figure 1. Survival curve by cluster. ... 9

Supplementary Figure 2. Schoenfeld residuals for the crude Cox-proportional hazard model. ... 10

For peer review only - http://bmjopen.bmj.com/site/about/guidelines.xhtml

For peer review only

Supplementary Methods

All the analyses were conducted with R and Python. The codes, either R scripts or Python Jupyter notebooks, are available as supplementary materials together with this manuscript.

Data preparation

We used individual-level data from the National Health and Nutrition Examination Survey (NHANES) III conducted between 1988-1994. All datasets were downloaded from the NHANES website on March 3rd, 2022; the mortality dataset was download on May 27th, 2022. The authors did not have privileged access. Data were pre-processed with R (NHANESiii_extraction.R). We used these datasets: adult.dat.txt (n=20,050); lab.sas.txt (n=29,314); exam.sas.txt (n=31,311); examdr.sas.txt (n=30,818); and HGUHS (n=14,797). The adult dataset was used as the prime; that is, the other datasets were merged to the adult dataset: adult + lab (n=20,050); + exam (n=20,050); + examdr (n=20,050); + HGUHS (n=20,050). We kept only the variables of interest, checked each variable, and recoded them as needed; for example, 9999 or equivalent values were set to missing, and 2 were coded to 0 when referred to 'no'. We merged the mortality dataset (n=33,994) with the pooled dataset and the sample size was kept at 20,050 (i.e., the pooled dataset was used as the prime) with 79 variables.

Study sample

We followed these criteria to select the study sample (Flowchart 1). First, we only included people in the age range from 20 to 74 (inclusive) years. Second, we only kept observations with hepatic imaging data. Third, we excluded people with positive evidence of Hepatitis B (HBsAG) or Hepatitis C (antiHCV). Fourth, we excluded people

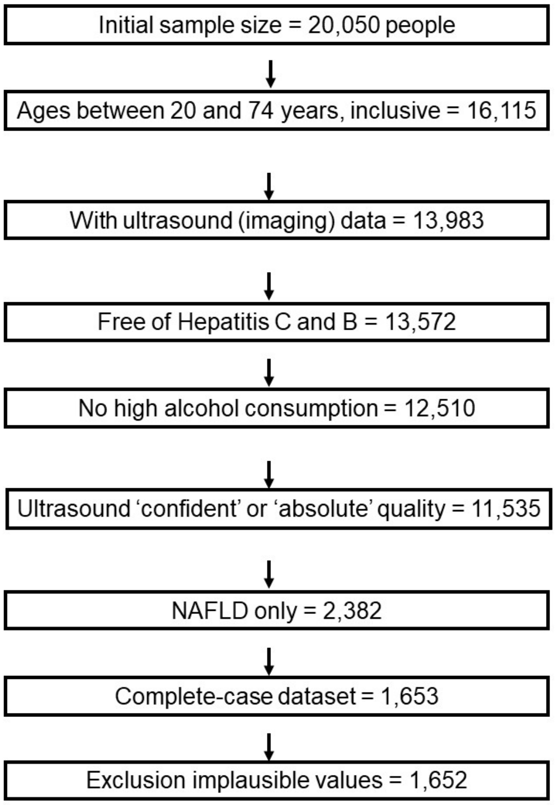

Flowchart 1. Study population.

For peer review only - http://bmjopen.bmj.com/site/about/guidelines.xhtml

with high alcohol consumption. We used two questions to define high alcohol consumption: i) *In the past 12 months, how many days of the year did you drink any alcoholic beverages?* and ii) *On the average, on the days that you drank alcohol, how many drinks did you have a day?* The answers to the second question were divided by 365.25 to compute the average number of drinks per day, which was then multiplied by the first question (how many days they drunk alcohol) to compute the number of daily alcohol drinks in the last year. For example, a person who drank alcoholic beverages 52 days in the last year, and on each occasion they drank three beverages, their daily consumption would be 52 x 3/365.25 = 0.43 alcoholic beverages per day in the last year. Men with more than two and women with more than one alcoholic beverages per day in the last year were excluded. Fifth, we only included observations which the hepatic imaging was deemed 'confident' or 'absolute'; this, to secure the highest quality of the outcome of interest (NAFLD); in addition, we only include people whose hepatic imaging revealed hepatic steatosis 'moderate-severe'. Sixth, we only kept the 10 predictors of interest and dropped all missing observations; in other words, we were to conduct a complete-case analysis. Seventh, we excluded observations outside the following plausibility ranges to secure high-quality data: BMI below and above 10 kg/m^2 and 80 kg/m^2; waist circumference below and above 30 cm and 200 cm; systolic blood pressure below and above 70 mmHg an 270 mmHg; fasting plasma glucose below and above 45 mg/dL and 540 mg/dL; total cholesterol below and above 20 mg/dL and 773 mg/dL; and triglycerides below and above 17 mg/dL and 1771 mg/dL. Finally, we included 1,652 observations in the analysis. For further details about this selection process please refer to the Jupyter notebook 1.Cleaning_data.ipynb.

Number of clusters

Selecting the *ideal* number of clusters in an unsupervised machine learning model is informed by both the data and expert knowledge. In here, we describe in detail the process we followed to reach the final number of clusters used in the analysis. Details about the analytical steps are presented in the Jupyter notebooks presented along the paper (2.Number_clusters.ipynb).

For peer review only - http://bmjopen.bmj.com/site/about/guidelines.xhtml

For peer review only

First, we displayed a dendrogram with Euclidean distances; this plot suggested there were five clusters. Of note, two of the six clusters grouped (very) few observations. Second, we displayed the Elbow plot (Figure 1) for one through 10 clusters. The ideal number of clusters would be that after which the Cost function does not change substantially and is the smallest. The table below shows the Cost function for each number of clusters, and the absolute arithmetic difference between two consecutive Cost functions. According to these figures (Table 1), the optimal number of clusters could be between five and six clusters; moreover, five clusters appeared to be *ideal* because the cost function was smallest and almost constant (~1.4) thereafter.

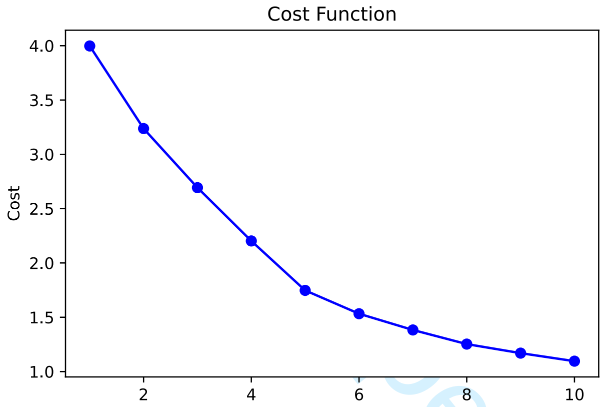

Figure 2. Elbow plot.

Table 1. Cost function for each number of cluster (i.e., elbow plot)

Number of clusters	Cost function	Difference with the immediate before
1	3.9975786924939500	
2	3.2371647353902400	-0.76
3	2.6931986798721200	-0.54
4	2.2026180284390100	-0.49
5	1.7472952094553600	-0.46
6	1.5326884453790900	-0.21
7	1.3831729448463400	-0.15
8	1.2530783891239300	-0.13
9	1.1698248951603600	-0.08
10	1.0958516180666800	-0.07

Third, we displayed the Silhouette plots and computed the Silhouette scores between two and 10 clusters (Table below). The number of clusters which Silhouette score is closest to 1 is preferred. According to these

For peer review only - http://bmjopen.bmj.com/site/about/guidelines.xhtml

figures (Table 2), two clusters would be *ideal* (i.e., highest Silhouette score), closely followed by three and four clusters.

Table 2. Silhouette score for each number of clusters.

Number of clusters	Silhouette scores
2	0.54608205593638
3	0.49123277407391
4	0.47822718686785
5	0.29615494816696
6	0.30743082083024
7	0.24963726715962
8	0.26441204421507
9	0.25048383421862
10	0.25036003409133

Fourth, we calculated the Jaccard index for three, four and five clusters (Table 3). So far, three, four and five clusters appear to be the best options. Jaccard scores ≥0.80 suggest good reproducibility of the cluster and would thus be preferred. According to these figures (Table below), three clusters had the highest Jaccard scores, all of which were ≥0.88. The Jaccard analysis was conducted in R (2.1.Jaccard.R).

Table 3. Jaccard score for each number of clusters from three to five clusters.

	**1 cluster**	**2 clusters**	**3 clusters**		
Jaccard bootstrap	0.8853153	0.9666082	0.9000518		
Dissolved	284	0	309		
Recovered	4402	4826	4579		
	**1 cluster**	**2 clusters**	**3 clusters**	**4 clusters**	
Jaccard bootstrap	0.5858137	0.8741936	0.5433940	0.6849515	

For peer review only - http://bmjopen.bmj.com/site/about/guidelines.xhtml

Dissolved	2564	0	2801	720	
Recovered	1728	4705	1279	1691	
	**1 cluster**	**2 clusters**	**3 clusters**	**4 clusters**	**5 clusters**
Jaccard bootstrap	0.7489208	0.7477933	0.5354848	0.5248099	0.6352783
Dissolved	1244	17	2839	2834	1918
Recovered	2942	2231	915	1139	1905

Fifth, exploratory analyses with five and six clusters showed that some clusters had (very) few observations and there were not unique profiles to characterize these clusters. Having four clusters was later discarded because of the Jaccard scores (see Table above). In conclusion, we chose three clusters as the *ideal* number of clusters for the analysis, which is also supported by the Jaccard score and (rather) consistent with the Elbow plot (Figure 1) and the Silhouette score (Table 2).

Of note, in exploratory analyses, we also tried selecting the number of clusters based on other algorithms and the results were consistent with those herein summarized; the algorithms we tried were: Balanced Iterative Reducing and Clustering using Hierarchies (BIRCH); spectral; and agglomerative.

For peer review only - http://bmjopen.bmj.com/site/about/guidelines.xhtml

Supplementary Table 1. Underlying characteristics of each cluster.

Cluster	Age		Body mass index (km/m^2)		Waist Circumference (cm)		Systolic blood pressure (mmHg)		Fasting plasma glucose (mg/dL)		Total cholesterol (mg/dL)		Triglycerides (mg/dL)		Aspartate Aminotransferase (U/L)		Alanine Aminotransferase (U/L)		Gamma-glutamyl transpeptidase (U/L)	
	Mean	SD	Mean	SD	Mean	SD	Mean	SD	Mean	SD	Mean	SD	Mean	SD	Mean	SD	Mean	SD	Mean	SD
Average	46.6	15.9	29.9	6.4	100.0	15.7	127.9	19.6	99.3	18.3	205.9	42.6	149.7	72.7	23.6	14.1	21.9	17.1	33.7	28.0
Lipid-Liver	47.1	13.9	30.2	4.7	103.3	11.5	132.5	18.3	114.9	43.1	241.0	55.8	495.7	221.1	34.6	40.5	36.5	44.2	93.0	133.2
Anthro-BP-Glucose	55.3	12.1	31.7	6.7	106.1	13.8	135.9	19.5	288.4	72.2	232.9	64.0	248.0	147.7	20.7	14.8	22.4	17.9	48.0	51.5

SD: standard deviation.

For peer review only - http://bmjopen.bmj.com/site/about/guidelines.xhtml

For peer review only

Supplementary Table 2. Pair-wise comparisons between clusters.

Predictor	Grupo 1	Grupo 2	Sample in Group 1	Sample in Group 2	Statistic	Degrees of freedom	p-value	Bonferroni-adjusted p-value
Age	Average	Lipid-Liver	1376	171	-0.4341	228.8684	6.650E-01	1.000E+00
	Average	Anthro-BP-Glucose	1376	105	-6.9071	132.8967	0.000E+00	0.000E+00
	Lipid-Liver	Anthro-BP-Glucose	171	105	-5.1494	243.0031	0.000E+00	0.000E+00
Body Mass Index	Average	Lipid-Liver	1376	171	-0.8983	254.9349	3.700E-01	1.000E+00
	Average	Anthro-BP-Glucose	1376	105	-2.7146	118.85	8.000E-03	2.400E-02
	Lipid-Liver	Anthro-BP-Glucose	171	105	-1.9792	167.1476	4.900E-02	1.470E-01
Waist Circumference	Average	Lipid-Liver	1376	171	-3.3914	256.2415	8.000E-04	2.400E-03
	Average	Anthro-BP-Glucose	1376	105	-4.3361	125.4833	0.000E+00	1.000E-04
	Lipid-Liver	Anthro-BP-Glucose	171	105	-1.7477	190.4266	8.200E-02	2.460E-01
Systolic Blood Pressure	Average	Lipid-Liver	1376	171	-3.0838	221.5132	2.000E-03	6.000E-03
	Average	Anthro-BP-Glucose	1376	105	-4.0781	120.6513	1.000E-04	2.000E-04
	Lipid-Liver	Anthro-BP-Glucose	171	105	-1.4565	209.4584	1.470E-01	4.410E-01
Plasma Glucose	Average	Lipid-Liver	1376	171	-4.6764	177.7001	0.000E+00	0.000E+00
	Average	Anthro-BP-Glucose	1376	105	-26.7622	105.0216	0.000E+00	0.000E+00
	Lipid-Liver	Anthro-BP-Glucose	171	105	-22.3014	150.0289	0.000E+00	0.000E+00
Total Cholesterol	Average	Lipid-Liver	1376	171	-7.9482	195.336	0.000E+00	0.000E+00
	Average	Anthro-BP-Glucose	1376	105	-4.2568	111.1312	0.000E+00	1.000E-04
	Lipid-Liver	Anthro-BP-Glucose	171	105	1.0709	197.489	2.860E-01	8.580E-01
Triglycerides	Average	Lipid-Liver	1376	171	-20.3273	174.601	0.000E+00	0.000E+00
	Average	Anthro-BP-Glucose	1376	105	-6.7507	107.88	0.000E+00	0.000E+00
	Lipid-Liver	Anthro-BP-Glucose	171	105	11.1491	272.009	0.000E+00	0.000E+00
Aspartate	Average	Lipid-Liver	1376	171	-3.521	175.1681	5.000E-04	1.600E-03
	Average	Anthro-BP-Glucose	1376	105	1.9415	118.8264	5.500E-02	1.650E-01
	Lipid-Liver	Anthro-BP-Glucose	171	105	4.0638	234.143	1.000E-04	2.000E-04
Alanine	Average	Lipid-Liver	1376	171	-4.2566	176.3829	0.000E+00	1.000E-04
	Average	Anthro-BP-Glucose	1376	105	-0.2754	118.8691	7.840E-01	1.000E+00
	Lipid-Liver	Anthro-BP-Glucose	171	105	3.683	244.7561	3.000E-04	9.000E-04
Glutamyl	Average	Lipid-Liver	1376	171	-5.8033	171.8756	0.000E+00	0.000E+00
	Average	Anthro-BP-Glucose	1376	105	-2.8266	108.7452	6.000E-03	1.800E-02
	Lipid-Liver	Anthro-BP-Glucose	171	105	3.9527	239.6927	1.000E-04	3.000E-04

For peer review only

Supplementary Figure 1. Survival curve by cluster.

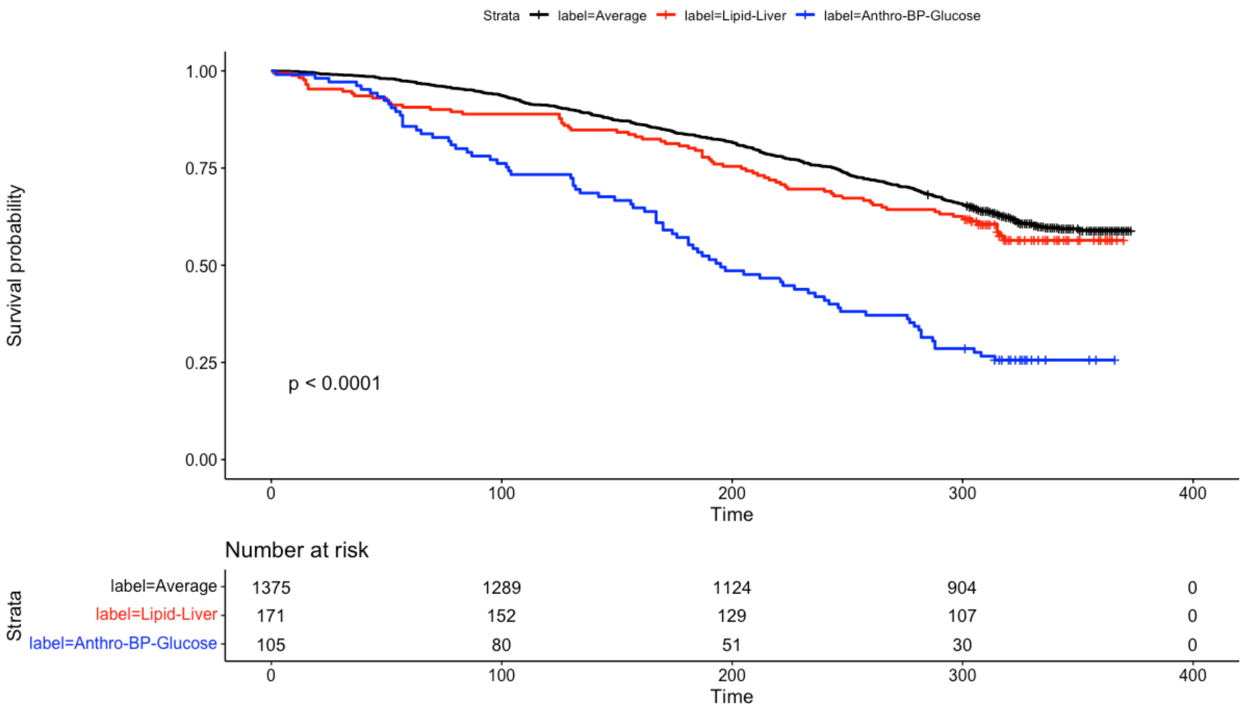

For peer review only

Supplementary Figure 2. Schoenfeld residuals for the crude Cox-proportional hazard model.

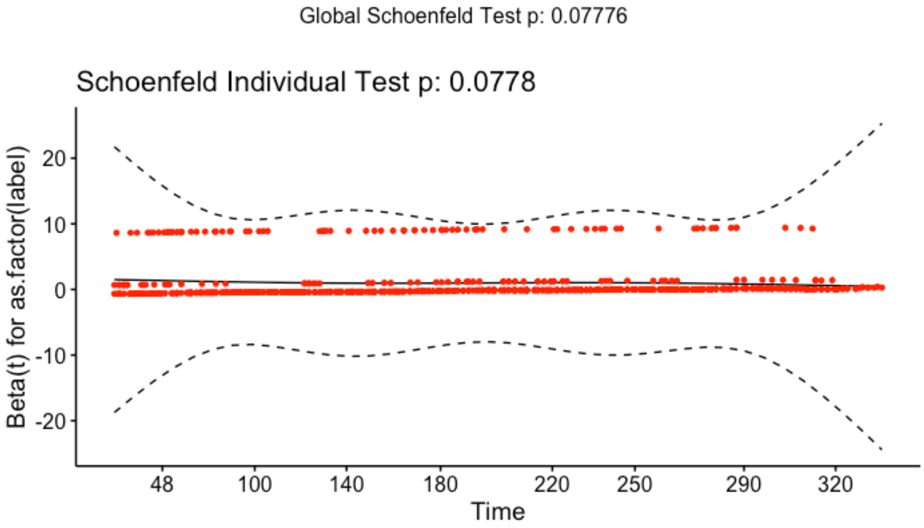

For peer review only - http://bmjopen.bmj.com/site/about/guidelines.xhtml

Phenotypes of non-alcoholic fatty liver disease (NAFLD) and all-cause mortality: Unsupervised machine learning analysis of NHANES III

Journal:	*BMJ Open*
Manuscript ID	bmjopen-2022-067203.R1
Article Type:	Original research
Date Submitted by the Author:	24-Oct-2022

Complete List of Authors:	Carrillo-Larco, Rodrigo; Imperial College London<br>Guzman-Vilca, Wilmer Cristobal; Universidad Peruana Cayetano Heredia; Universidad Peruana Cayetano Heredia,<br>Castillo-Cara, Manuel; Universidad Politécnica de Madrid, Artificial Intelligence Department<br>Alvizuri-Gómez, Claudia; Hospital Nacional Cayetano Heredia, Department of Gastroenterology<br>Alqahtani, Saleh; Johns Hopkins University, Department of Medicine<br>Garcia Larsen, Vanessa; Johns Hopkins University Bloomberg School of Public Health, Department of International Health

**Primary Subject Heading**:	Epidemiology
Secondary Subject Heading:	Epidemiology
Keywords:	GASTROENTEROLOGY, EPIDEMIOLOGY, PREVENTIVE MEDICINE, PUBLIC HEALTH

SCHOLARONE™
Manuscripts

I, the Submitting Author has the right to grant and does grant on behalf of all authors of the Work (as defined in the below author licence), an exclusive licence and/or a non-exclusive licence for contributions from authors who are: i) UK Crown employees; ii) where BMJ has agreed a CC-BY licence shall apply, and/or iii) in accordance with the terms applicable for US Federal Government officers or employees acting as part of their official duties; on a worldwide, perpetual, irrevocable, royalty-free basis to BMJ Publishing Group Ltd ("BMJ") its licensees and where the relevant Journal is co-owned by BMJ to the co-owners of the Journal, to publish the Work in this journal and any other BMJ products and to exploit all rights, as set out in our licence.

The Submitting Author accepts and understands that any supply made under these terms is made by BMJ to the Submitting Author unless you are acting as an employee on behalf of your employer or a postgraduate student of an affiliated institution which is paying any applicable article publishing charge ("APC") for Open Access articles. Where the Submitting Author wishes to make the Work available on an Open Access basis (and intends to pay the relevant APC), the terms of reuse of such Open Access shall be governed by a Creative Commons licence – details of these licences and which Creative Commons licence will apply to this Work are set out in our licence referred to above.

Other than as permitted in any relevant BMJ Author's Self Archiving Policies, I confirm this Work has not been accepted for publication elsewhere, is not being considered for publication elsewhere and does not duplicate material already published. I confirm all authors consent to publication of this Work and authorise the granting of this licence.

For peer review only

Phenotypes of non-alcoholic fatty liver disease (NAFLD) and all-cause mortality: Unsupervised machine learning analysis of NHANES III

Rodrigo M Carrillo-Larco [1,2,3]

Wilmer Cristobal Guzman-Vilca [2,4,5]

Manuel Castillo-Cara [6]

Claudia Alvizuri-Gómez [7]

Saleh A Alqahtani [8,9]

Vanessa García-Larsen [10]

1. Department of Epidemiology and Biostatistics, School of Public Health, Imperial College London, London, United Kingdom.
2. CRONICAS Centre of Excellence in Chronic Diseases, Universidad Peruana Cayetano Heredia, Lima, Peru.
3. Universidad Continental, Lima, Peru.
4. School of Medicine Alberto Hurtado, Universidad Peruana Cayetano Heredia, Lima, Peru.
5. Sociedad Científica de Estudiantes de Medicina Cayetano Heredia (SOCEMCH), Universidad Peruana Cayetano Heredia, Lima, Peru.
6. Ontology Engineering Group, Artificial Intelligence Department, Universidad Politécnica de Madrid, Madrid, Spain.
7. Department of Gastroenterology, Hospital Nacional Cayetano Heredia. Lima, Peru.
8. King Faisal Specialist Hospital & Research Centre, Riyadh 11564, Saudi Arabia.
9. Division of Gastroenterology and Hepatology, Department of Medicine, Johns Hopkins University, Baltimore, MD 21205, USA.
10. Program in Human Nutrition, Department of International Health, The Johns Hopkins Bloomberg School of Public Health, Baltimore, MD 21205, USA.

Corresponding Author:

Vanessa García-Larsen

Email: vgla@jhu.edu

For peer review only

For peer review only - http://bmjopen.bmj.com/site/about/guidelines.xhtml

ABSTRACT

Objectives: Non-alcoholic fatty liver disease (NAFLD) is a non-communicable disease with a rising prevalence worldwide and with large burden for patients and health systems. To date, the presence of unique phenotypes in NAFLD patients has not been studied, and their identification could inform precision medicine and public health with pragmatic implications in personalized management and care for NAFLD patients.

Design: Cross-sectional and prospective (up to December 31st, 2019) analysis of NHANES III (1988-1994).

Primary and secondary outcomes measures: NAFLD diagnosis was based on liver ultrasound. The following predictors informed an unsupervised machine learning algorithm (k-means): body mass index; waist circumference; systolic blood pressure (SBP); plasma glucose; total cholesterol; triglycerides; liver enzymes alanine aminotransferase, aspartate aminotransferase, and gamma glutamyl transferase. We summarized (means) and compared the predictors across clusters. We used Cox Proportional Hazard models to quantify the all-cause mortality risk associated with each cluster.

Results: 1,652 patients with NAFLD (mean age 47.2 years and 51.5% women) were grouped into three clusters: Anthro-SBP-Glucose (6.36%; highest levels of anthropometrics, SBP and glucose); Lipid-Liver (10.35%; highest levels of lipid and liver enzymes); and Average (83.29%; predictors at average levels). Compared to the Average phenotype, the Anthro-SBP-Glucose phenotype had higher all-cause mortality risk (aHR=2.88; 95% CI: 2.26-3.67); the Lipid-Liver phenotype was not associated with higher all-cause mortality risk (aHR=1.11; 95% CI: 0.86-1.42).

Conclusions: There is heterogeneity in NAFLD patients, whom can be divided into three phenotypes with different mortality risk. These phenotypes could guide specific interventions and management plans, thus advancing precision medicine and public health for NAFLD patients.

Keywords: data-driven; artificial intelligence; non-communicable diseases; precision medicine.

For peer review only

Strengths and limitations of this study

- We leveraged on population-based data with image-based NAFLD diagnosis and long-term follow-up.
- We applied machine learning techniques to identify phenotypes amongst people with NAFLD, consistent with precision medicine and precision public health.
- Arguably, the gold standard for NAFLD diagnosis is liver biopsy, though given the source of the dataset (population-based nationally representative study), taking biopsies was not possible.
- We did not use diet-related variables because measuring these may vary between places and observers unlike blood biomarkers.

For peer review only

INTRODUCTION

The epidemiology of non-communicable diseases (NCDs) is largely driven by cardiometabolic risk factors and diseases, namely dyslipidaemias, type 2 diabetes mellitus (T2DM), hypertension, and cardiovascular diseases. Nonetheless, there are other NCDs rapidly growing along with, and as a consequence of,[1,2] the aforementioned cardiometabolic conditions. Non-alcoholic fatty liver disease (NAFLD) is an outstanding example with a soaring prevalence worldwide,[3-6] high economic costs for patients and health systems,[7,8] and poor patient-oriented outcomes.[9] Despite this high disease burden, there are no specific treatments for NAFLD other than managing the underlying conditions like obesity or T2DM together with recommendations for healthy lifestyles.[10-14] Consistent with the global call for precision medicine and public health, a way to maximize the benefits of available and forthcoming[15-17] treatments for NAFLD, could be to identify phenotypes in NAFLD patients. Therefore, specific management plans can be proposed according to the underlying profile of each phenotype. A data-driven machine learning (ML) approach has been proven to be a reliable method to identify phenotypes amongst patients, and there are several examples for T2DM.[18] However, NAFLD research has not yet used ML to identify phenotypes or subpopulations, even though it could signal groups of patients for which different management plans can be provided and different prognosis can be expected. We aimed to identify phenotypes in people with NAFLD in the general population and to quantify the all-cause mortality risk associated with each phenotype.

METHODS

Study design and data sources

This is a data-driven analysis following an unsupervised ML approach. We conducted a cross-sectional analysis whereby we identified clusters of NAFLD patients and described the underling phenotype of each cluster. Analysing the same study population, we also conducted a prospective analysis whereby we investigated whether the phenotypes were associated with higher all-cause mortality risk. Overall, for both the cross-sectional and prospective analyses, we used the same study population form the National Health and Nutrition Examination Survey (NHANES) III.

For peer review only - http://bmjopen.bmj.com/site/about/guidelines.xhtml

We used individual-level data from the NHANES III conducted between 1988-1994. The NHANES III included a nationally representative sample of non-institutionalized individuals in the United States of America (USA).[19] We used NHANESS III, and no more recent iterations of this survey, because NHANESS III is the only with liver ultrasound data which provide high-quality information for NALFD research.

Study population

The study population included people aged between 20 and 74 years who had hepatic imaging data. Hepatic imaging refers to ultrasound examinations conducted following standard procedures to secure consistent and reliable results for all participants.[20] We included people whose imaging results were deemed "confident" or "absolute" to secure high-quality data for the definition of NAFLD.[20] For the analyses, we only included people with NAFLD defined as a "moderate-severe" hepatic steatosis.[20] In so doing, we excluded missing observations in liver ultrasound data; missing patterns in this variable were not explored.

People with a positive test for Hepatitis B (HBsAG) and Hepatitis C (antiHCV) were excluded, and so were people with high alcohol consumption (Supplementary Materials p. 02). People whose risk factors levels were below or above these plausibility thresholds were excluded too: body mass index (BMI) between 10 kg/m^2 and 80 kg/m^2; waist circumference between 30 cm and 200 cm; systolic blood pressure (SBP) between 70 mmHg and 270 mmHg; plasma glucose between 45 mg/dL and 540 mg/dL; total cholesterol between 20 mg/dL and 773 mg/dL; and triglycerides between 17 mg/dL and 1,771 mg/dL. Details about data preparation and selection of the study population are available in Supplementary Materials p. 02.

Variables

From the NHANES dataset we selected ten predictors, which will inform the composition of the clusters and characterize the phenotype of each cluster. These predictors were selected because they are established cardiometabolic risk factors, and cardiovascular diseases are a leading cause of mortality in people with NAFLD.[21] We also included liver biomarkers as predictors because NAFLD is a chronic liver condition. The predictors included chronological age (years); BMI (kg/m^2) based on measured weight and height; waist circumference (cm); SBP (mmHg); plasma glucose (mg/dL) which was used regardless of fasting duration

For peer review only - http://bmjopen.bmj.com/site/about/guidelines.xhtml

(41.53% or 686/1,652 had a fasting duration <8 hours) to maximize sample size for the study population comprising only people with NAFLD; total cholesterol (mg/dL); triglycerides (mg/dL); serum alanine aminotransferase (U/L); serum aspartate aminotransferase (U/L); and serum gamma glutamyl transferase (U/L; GGT). All blood biomarkers where collected and analysed following standard procedures.[22] We conducted a completed-case analysis for the ten predictors of interest.

We did not include more predictors, such as coagulation or anaemia biomarkers, to deliver a model that can be replicated in many places and by researchers who may not have access to more sophisticated biomarkers. Including more or more sophisticated predictors would limit the use of our model to places where these predictors are available. This would, most likely, exclude several primary healthcare facilities and those in rural or resource-limited settings.

Mortality data are also available for NHANES III.[23] The National Centre for Health Statistics (NCHS) delivers a mortality dataset linked to NHANES participants (adults only). Follow-up duration, in person months, is computed from the examination date to the date of death or censorship (December 31st, 2019). Because a priori we did not know the number of clusters or their underlying profiles, we focused on all-cause mortality rather than on a specific cause of death. For example, had the unsupervised analysis suggested many clusters, it would have compromised the confidence intervals from the regressions if the exposure variable was divided in several clusters or groups.

Analysis

Open-access resources

The analyses were conducted in Python 3.10 using PyCharm 2021.3.3. and Jupyter Notebooks; we also used R 4.1.2 for the statistical analyses. The Jupyter Notebooks and R scripts are available as Supplementary Materials. All analytical methods are detailed in Supplementary Materials pp. 02-05.

For peer review only - http://bmjopen.bmj.com/site/about/guidelines.xhtml

Unsupervised machine learning analysis

We applied principal components analysis (PCA) to the ten predictors. We chose four principal components because these explained 95.19% of the variance. In other words, we retained 95.19% of the information in the original dataset with these four components. On these four principal components, we applied the k-means algorithm, an unsupervised ML method, to identify clusters of NAFLD patients with unique phenotypes. To define the number of clusters, which is unknown at the beginning of any unsupervised ML analysis, we followed a data-driven approach together with expert knowledge about NAFLD. First, we plotted a dendrogram with Euclidean distances, which suggested to use five clusters. Second, the Elbow method suggested to use five or six clusters. Third, the Silhouette score was highest with two clusters, followed by three clusters. Fourth, the Jaccard score was highest for three clusters. Therefore, based on the above metrics and expert knowledge, we decided on three clusters. In exploratory analyses we also tried selecting the number of clusters based on other algorithms and the results were consistent with those herein summarized; the other algorithms we tried were: Balanced Iterative Reducing and Clustering using Hierarchies (BIRCH); spectral; and agglomerative.

Statistical analysis

First, we conducted a descriptive analysis to characterize the three clusters in terms of the ten predictors. That is, we summarized the mean of the ten predictors in each cluster and used Boxplots. We used t-tests for pair-wise comparisons between all clusters; the p-value is reported accounting for the Bonferroni correction for multiple comparisons.

Second, to study the all-cause mortality associated with each cluster, we ran Cox Proportional Hazard models reporting Hazard Ratios (HR) along with the 95% Confidence Interval (95% CI). We used time in the study (difference between when the one-off baseline evaluation occurred and the endpoint) as the time scale in the Cox models. We fitted three models: i) crude model only including the outcome (all-cause mortality) and the exposure (clusters, categorical variable with three levels); ii) model 1, including sex; and iii) model 2, including sex, smoking status (current smoker of cigarettes, no vs yes), years of education, and household income (<20,000$ vs ≥20,000$ in the last year). The proportional hazard assumption was

For peer review only - http://bmjopen.bmj.com/site/about/guidelines.xhtml

verified in the crude model, and we did not observe evidence that this assumption was violated. We did not adjust for age or other cardio-metabolic risk factors because these were included in the clusters which are the main predictors (i.e., independent variable) in the regression models. Together with the Cox regression model, we also report the absolute number of deaths per group, the total follow-up duration per group, as well as the incidence rate per 1,000 person-years which was computed with the epi.conf function from the epiR package in R.

The statistical analysis did not account for the complex survey design of the NHANES survey (e.g., sampling weights). We aimed to characterize the clusters and study if they were associated with higher all-cause mortality risk, rather than to provide prevalence estimates or means that are representative at the national level.

For peer review only

Sensitivity analysis

We carried several sensitivity analyses to elucidate if any observed differences were driven by the fact that we did not only include people with more than eight hours of fasting. First, we compared the mean plasma glucose levels across phenotypes restricting the sample to those with eight or more hours of fasting duration. Second, we ran the fully adjusted Cox model (model 2) restricting the sample to those with eight or more hours of fasting. Third, we ran the fully adjusted Cox model (model 2) including fasting duration (in hours) as an additional confounder rather than restricting the sample to those with eight or more hours of fasting duration. To explore whether the phenotypes were associated with all-cause mortality above and beyond the individual risk factors, we also ran model 2 including the 10 predictors as confounders.

Role of the funding source

This study received no funding. The opinions on this document are those from the authors alone and do not represent the views of the institutions to which they belong. The first two authors collated, pooled, and analysed the data; the first two authors vouch for the accuracy of the results.

For peer review only - http://bmjopen.bmj.com/site/about/guidelines.xhtml

Patient and public involvement

It was not appropriate or possible to involve patients or the public in the design, or conduct, or reporting, or dissemination plans of our research.

Ethics

We analyzed individual-level open access data. Human subjects were not directly involved in this work. This work was deemed as of minimal risk. We did not seek approval by an ethics committee because we analyzed de-identified data in the public domain available for independent reanalyzes.

RESULTS

Study population

There were 1,652 people with NAFLD, the mean age was 47.2 years and 51.5% were women. Across the study population the mean BMI was 30.0 kg/m^2 and the mean waist circumference was 100.7 cm; the mean SBP was 128.9 mmHg, the mean plasma glucose was 112.9 mg/dL, the mean total cholesterol was 211.3 mg/dL, and the mean triglycerides was 191.8 mg/dL; the mean aspartate was 24.5 U/L, the mean alanine was 23.5 U/L and the mean GGT was 40.7 U/L.

Cluster profiles

Given the underlying profiles of each phenotype (Table 1 and Figure 1), these were labelled as it follows: Lipid-Liver (i.e., highest levels of cholesterol and liver enzymes); Anthro-SBP-Glucose (i.e., highest levels of anthropometrics, SBP and glucose); and Average (i.e., mean levels of the predictors were always in between the former two phenotypes). The Average phenotype grouped 1,376 people (83.29%), followed by the Lipid-Liver (171 people, 10.35%), and the Anthro-SBP-Glucose phenotype with 105 people (6.36%).

For peer review only - http://bmjopen.bmj.com/site/about/guidelines.xhtml

For peer review only

Table 1. General characteristics of participants by phenotype cluster

Clusters	Age	BMI	Waist	SBP	Glucose	TC	Triglycerides	Aspartate	Alanine	GGT
Average	46.6 (15.9)	29.9 (6.4)	100.0 (15.7)	127.9 (19.6)	99.3 (18.3)	205.9 (42.6)	149.7 (72.7)	23.6 (14.1)	21.9 (17.1)	33.7 (28.0)
Lipid-Liver	47.1 (13.9)	30.2 (4.7)	103.3 (11.5)	132.5 (18.3)	114.9 (43.1)	241.0 (55.8)	495.7 (221.1)	34.6 (40.5)	36.5 (44.2)	93.0 (133.2)
Anthro-SBP-Glucose	55.3 (12.1)	31.7 (6.7)	106.1 (13.8)	135.9 (19.5)	288.4 (72.2)	232.9 (64.0)	248.0 (147.7)	20.7 (14.8)	22.4 (17.9)	48.0 (51.5)

All variables are expressed as means (standard deviation). Comparisons across clusters are shown in Figure 1 and Supplementary Table 1. BMI: body mass index (kg/m^2); waist refers to waist circumference (cm); SBP: systolic blood pressure (mmHg); glucose refers to plasma glucose (mg/dL); TC: total cholesterol (mg/dL); triglycerides are expressed in mg/dL; aspartate refers to aspartate aminotransferase (AST; U/L); alanine refers to alanine aminotransferase (ALT; U/L); GGT: Gamma-glutamyl transferase (U/L)

For peer review only

Differences between the Average and the Lipid-Liver phenotypes were always statistically significant, except for age (p=1) and BMI (p=1) (Figure 1 and Supplementary Table 1). Differences between the Average and Anthro-SBP-Glucose phenotypes were always statistically significant except for alanine (p=1) and aspartate (p=0.165). Differences between the Lipid-Liver phenotype and the Anthro-SBP-Glucose phenotype were significant for six predictors: age (older in the Anthro-SBP-Glucose); plasma glucose (higher in the Anthro-SBP-Glucose); triglycerides (higher in the Lipid-Liver); alanine (higher in the Lipid-Liver); aspartate (higher in the Lipid-Liver); and GGT (higher in the Lipid-Liver). That is, most of the unique features of the Lipid-Liver phenotype were different –higher– in comparison to the Anthro-SBP-Glucose phenotype; conversely, only glucose in the Anthro-SBP-Glucose phenotype was significantly different –higher– relative to the Lipid-Liver phenotype.

All-cause mortality

Amongst those who died, 78.0% were in the Average phenotype; 10.5% were in the Anthro-SBP-Glucose phenotype, and 11.5% were in the Lipid-Liver phenotype. The mean follow-up duration was 270.1 months (~22.5 years), ranging from a month to 373 months. The survival probability decreased much faster for the Anthro-SBP-Glucose phenotype than for the Lipid-Liver phenotype (Supplementary Figure 1).

The crude Cox proportional hazard model showed that, in comparison to the Average phenotype, the Lipid-Liver was not associated with higher all-cause mortality (HR=1.17; p=0.217); conversely, the Anthro-SBP-Glucose phenotype were associated with higher all-cause mortality risk (HR=2.94; p<0.001; Table 2). After adjusting for sex, the association for the Lipid-Liver phenotype was still not significant (HR=1.11; p=0.431), and the association for the Anthro-SBP-Glucose phenotype remained significant (HR=2.97; p<0.001). The same pattern was observed when we further adjusted for smoking, education, and household income whereby the Lipid-Liver phenotype was not associated with higher all-cause mortality risk (HR=1.21; p=0.427), yet the Anthro-SBP-Glucose phenotype was still strongly associated with higher all-cause mortality risk (HR=2.88; p<0.001).

For peer review only - http://bmjopen.bmj.com/site/about/guidelines.xhtml

Table 2. Mortality risk in NAFLD patients by phenotype cluster

Cluster	Absolute number of deaths	Total follow-up duration (years)	Incidence rate per 1,000 person-years (95% CI)	Outcome: all-cause mortality (HR, 95% CI)*		
				**Crude** N=1,651 Events = 678	**Model 1** N=1,651 Events = 678	**Model 2** N=1,622 Events = 663
Average	529	31,707.083	16.7 (15.3-18.2)	1	1	1
Lipid-Liver	71	3,685.417	19.3 (15.1-24.3)	1.17 (0.91-1.50)	1.11 (0.86-1.42)	1.11 (0.86-1.42)
Anthro-SBP-Glucose	78	1,766.000	44.2 (34.9-55.1)	2.94 (2.32-3.74)	2.97 (2.34-3.78)	2.88 (2.26-3.67)

In the Average group, there was one observation with missing data on follow-up duration. *Cox proportional hazard ratios; Model 1 included sex. Model 2 included, in addition to sex, smoking status (current, no vs yes); years of education (numeric variable); and household income (<20,000$ vs ≥20,000$ in the last year). For the crude model, the global test for the proportional-hazard assumption was not significant (p=0.078), suggesting this assumption holds which is also supported by the Schoenfeld residuals plot (Supplementary Figure 2). The exposure variable for all models (crude, model 1, and model 2) was a three-level variable derived from the cluster analysis; in other words, the cluster analysis derived a new variable with three levels which we called phenotypes (Average, Lipid-Liver, and Anthro-SBP-Glucose) whose profiles were described in Figure 1. Neither of the three models included the predictors used to derive the clusters or phenotypes. The predictors were used to derive and characterize the phenotypes only (not to adjust the models).

For peer review only

Sensitivity analyses

The Anthro-SBP-Glucose phenotype still had the highest mean plasma glucose (273.5 mg/dL), followed by the Lipid-Liver phenotype (115.9 mg/dL), and the Average phenotype (101.2 mg/dL). The p-value for the Bonferroni-adjusted t-test for all pairwise comparisons was significant (p<0.003).

When model 2 for the Cox regression was restricted to people with eight or more hours of fasting, the results were consistent with the main analysis: the Anthro-SBP-Glucose phenotype was strongly associated with higher all-cause mortality risk (HR=2.65; 95% CI: 1.90-3.70; p<0.001), and the association for the Lipid-Liver phenotype was not significant (HR=1.12; 95% CI: 0.80-1.58; p=0.512). Virtually the same results were observed when model 2 included fasting duration as a covariate rather than restricting the sample to those with eight or more hours of fasting duration: HR=2.89 (95% CI: 2.27-3.69; p<0.001) for the Anthro-SBP-Glucose, and HR=1.11 (95% CI: 0.86-1.43; p=0.414) for the Lipid-Liver phenotype.

When model 2 also included the 10 predictors used to define the phenotypes, neither the Lipid-Liver (HR=1.20; 95% CI: 0.82-1.74; p=0.342) phenotype nor the Anthro-SBP-Glucose (HR=1.46; 95% CI: 0.90-2.38; p=0.126) phenotype was associated with higher all-cause mortality risk.

DISCUSSION

Main results

In a sample of NAFLD patients from the general population and using simple predictors, a ML analysis revealed three phenotypes with unique profiles. The Anthro-SBP-Glucose phenotype had the highest mean levels of BMI, waist circumference, SBP and plasma glucose. Patients in the Lipid-Liver phenotype had, on average, the highest levels of cholesterol and liver biomarkers. There was a third phenotype whose members had average risk factor levels. Most NAFLD patients belonged to the Average phenotype (83%), followed by the Lipid-Liver (10%) and Anthro-SBP-Glucose (6%) phenotypes. In comparison to the Average phenotype, the Anthro-SBP-Glucose phenotype had higher all-cause mortality risk; the all-cause mortality risk appeared not to be different between the Average and Lipid-Liver phenotypes.

For peer review only - http://bmjopen.bmj.com/site/about/guidelines.xhtml
For peer review only

We believe that these findings have the potential to advance precision medicine and public health by identifying variations in the clinical and metabolic presentation of NAFLD. Such variations suggest three homogenous phenotypes. NAFLD patients with the Anthro-SBP-Glucose phenotype, which was associated with the highest all-cause mortality risk, should receive urgent counselling and medication to reduce weight, SBP and glucose levels. While maintaining a healthy lifestyle should always be a cornerstone in the management of NAFLD patients, those with the Lipid-Liver phenotype could most benefit from novel therapies targeting cholesterol and liver physiology.[15-17] The Average phenotype was the most frequent. This signals a group of NAFLD patients who should be carefully monitored, though, perhaps, not with the same frequency or intensity as for the other phenotypes in which risk factors levels were higher and had higher all-cause mortality risk.

Results in context

NAFLD, defined as >5% of hepatic steatosis in images or histology in the absence of secondary causes (e.g., viral hepatitis),[11] is the most common cause of chronic liver disease in the last decade.[24] Although NAFLD is distributed worldwide, the prevalence of NAFLD is particularly high in the Middle East and South America.[24] Future research in these world regions could elaborate on our work to trial specific recommendations according to the patient's cluster.

To date, there are no specific treatments to prevent or cure NAFLD. For example, vitamin E has been proposed as a potential therapy for NAFLD, because it may improve non-alcoholic steatohepatitis. However, systematic reviews and meta-analysis of randomised trials have shown inconsistent evidence to recommend vitamin E for NAFLD;[25] moreover, potential adverse effects of vitamin E should be taken into consideration.[26] Although other vitamins may play a role as well, to the best of our knowledge, their effect has not been summarized in meta-analysis of randomized control trials (high level of evidence) and is less elaborated in clinical guidelines. Current strategies focus on weight loss, exercise. and diet. Mediterranean diet appears to reduce hepatic steatosis due to reduction in insulin-resistance and total lipid concentrations;[27] likewise, weight loss of ≥7% correlates with improvement of histological features.[26]

For peer review only - http://bmjopen.bmj.com/site/about/guidelines.xhtml

Overall, implementing these interventions to improve healthy lifestyles should be the standard of care for patients with NAFLD, and more so for those in the Anthro-SBP-Glucose phenotype herein found.

In 2020, an international consensus proposed the term *metabolic associated fatty liver disease* (MAFLD) to substitute NAFLD due to the strong metabolic component of this chronic liver condition.[28] Our work may support the MAFLD nomenclature because the Anthro-SBP-Glucose phenotype, largely consisting of traits consequence of metabolic dysfunction, had the highest all-cause mortality risk. Nevertheless, our work also signals there is heterogeneity in patients with NAFLD which may better guide treatment options above and beyond one specific nomenclature (NAFLD vs MAFLD).

Few studies have quantified the association between phenotypes and mortality in patients with NAFLD. Golabi *et al*,[29] evaluated the effect of abdominal and overall adiposity on the mortality risk in patients with NAFLD; after 22.4 years of follow-up, they observed that patients with NAFLD and normal BMI but high waist circumference, had higher risk of cardiovascular mortality.[29] This is consistent with the Anthro-SBP-Glucose phenotype having the highest all-cause mortality risk. It is worth noting that, even though on average the Anthro-SBP-Glucose phenotype had high levels of anthropometrics, SBP and glucose, it is not the same as a dichotomous variable whereby people with high anthropometrics, SBP and glucose were on one or the other category of the dichotomous variable (similar to the definition of metabolic syndrome); nevertheless, the additive effect of the anthropometrics, SBP and glucose should be noted and could potential explain the higher mortality.

Strengths and limitations

We leveraged on population-based data with image-based[20] NAFLD diagnosis and long-term follow-up.[23] We applied ML techniques to identify phenotypes amongst people with NAFLD, consistent with precision medicine and precision public health. Not only did we describe the underlying profile of each phenotype, but we also quantified the all-cause mortality risk associated with each phenotype.

For peer review only - http://bmjopen.bmj.com/site/about/guidelines.xhtml

For peer review only

Notwithstanding, there are limitations we should acknowledge. First, the gold standard to diagnose NAFLD requires a liver biopsy. Collecting a liver biopsy from a large population-based sample as that of NHANES would be (almost) impossible. From a clinical perspective, the fact that NAFLD was diagnosed with liver ultrasound could be a limitation; however, from a global or population health perspective, it could be a step forward to characterize phenotypes in people with NAFLD. Second, aiming to deliver a friendly and easy to replicate model, we included ten predictors that may be accessible to many clinicians and researchers. Had we used more sophisticated predictors, it would limit the reproducibility of our model by a broad audience. However, this decision could have led to the omission of other potential phenotypes. For example, if we had included anaemia biomarkers (e.g., haemoglobin or ferritin), there could have been an additional phenotype characterized by anaemia. Our work opens the door to use ML in NAFLD research aiming for precision medicine and public health, and future work could expand our set of phenotypes for specific setting where access to these predictors is not a limitation. In this line, although diet is a key variable in NAFLD, we did not include diet-based variables (including alcoholic beverages) in the cluster analysis; this, because, while inexpensive, collecting diet information may be troublesome, may require of specific tools (e.g., 24-hour diet recalls), and may need context-expert knowledge to ask about foods consumed locally. Similarly, physical activity was not included in the clustering analysis owing to the complexities to measure it. For example, whether physical activity is measured with metabolic equivalents (METs) or with questions about days/hours involved in physical activity, not to mention whether it was further disaggregated by moderate or vigorous activity. As argued before for more sophisticated biomarkers, including diet and physical activity profiles could also limit the applicability of the clusters because of the complexities and heterogeneity inherit to these lifestyle cardiometabolic risk factors. Third, we analysed data from NHANES III collected in the late 1980s and early 1990s; that is, data collected roughly 30 years ago. We used NHANES III only because it has liver ultrasound data unlike more recent iterations of the NHANES. Readers should interpret our findings and recommendations considering this limitation. Future work should replicate, verify, and advance our work with more contemporary data. Fourth, when missing observations were excluded (because we planned a complete-case analysis) we did not explore the missingness pattern which is customary in epidemiological research where, if necessary, multiple imputation is conducted and results account for Rubin's rules. The backbone of our work is the unsupervised machine learning analysis which

For peer review only

needs complete-case data. To the best of our knowledge, there are no techniques to conducted unsupervised machine learning analyses after multiple imputation. Fifth, with the focus of this work on the identification of unique phenotypes complemented by the mortality analysis, the Cox regressions were not enriched with additional analyses including cause-specific mortality, competing risks, effect modification or interactions. On one hand, some of these analyses would have benefitted from a much larger sample size; on the other hand, some of these analyses depend on both the exposure and outcome variables and in our case, the exposure variable (cluster) was a complex and heterogenous construct of multiple cardiometabolic biomarkers and liver enzymes.

Conclusions

There is heterogeneity in NAFLD patients, whom can be divided into three phenotypes. These phenotypes are either characterized by high levels of anthropometrics, SBP and glucose, or by high levels of cholesterol and liver biomarkers. These phenotypes could guide specific interventions, together with personalized management and follow-up plans thus advancing precision medicine and public health for NAFLD patients.

For peer review only

Contributions

RMC-L and VG-L conceived the research question. RMC-L pooled and processed the datasets with support from WCG-V. RMC-L conducted the machine learning analysis with support from MC-C. RMC-L conducted the statistical analysis. RMC-L drafted the first version of the manuscript with support from WCG-V, MC-C, CA-G, SAA and VG-L. WCG-V, MC-C, CA-G, SAA, and VG-L provided critical input, commented, and edited the manuscript. RMC-L, WCG-V, MC-C, CA-G, SAA, and VG-L approved the submitted version.

Data availability

All NHANES datasets are publicly available and can be download from the official website: https://www.cdc.gov/nchs/nhanes/index.htm All analysis code (Jupyter Notebooks and R Scripts) are available as Supplementary Materials.

For peer review only

Funding

RMC-L is supported by a Wellcome Trust International Training Fellowship (Wellcome Trust 214185/Z/18/Z).

Conflict of interests

None to declare.

Patients consent

Not applicable

Permission to reproduce materials from other sources

Not applicable

Clinical trial registration

Not applicable

For peer review only - http://bmjopen.bmj.com/site/about/guidelines.xhtml

FIGURES

Figure 1. Distribution of the predictors by phenotype cluster

P-values are for pair-wise comparisons and Bonferroni-adjusted for multiple comparisons (Supplementary Table 1). BMI: body mass index (kg/m^2); GGT: Gamma-glutamyl transferase (U/L).

For peer review only

For peer review only - http://bmjopen.bmj.com/site/about/guidelines.xhtml

Ethics

We analyzed open access data at the country level. Human subjects were not directly involved in this work.

This work was deemed as of minimal risk.

For peer review only

For peer review only - http://bmjopen.bmj.com/site/about/guidelines.xhtml

REFERENCES

1. Stefan N, Cusi K. A global view of the interplay between non-alcoholic fatty liver disease and diabetes. *The lancet Diabetes & endocrinology* 2022; **10**(4): 284-96.

2. Sarwar R, Pierce N, Koppe S. Obesity and nonalcoholic fatty liver disease: current perspectives. *Diabetes, metabolic syndrome and obesity : targets and therapy* 2018; **11**: 533-42.

3. Le MH, Yeo YH, Li X, et al. 2019 Global NAFLD Prevalence: A Systematic Review and Meta-analysis. *Clinical gastroenterology and hepatology : the official clinical practice journal of the American Gastroenterological Association* 2021.

4. Li J, Zou B, Yeo YH, et al. Prevalence, incidence, and outcome of non-alcoholic fatty liver disease in Asia, 1999-2019: a systematic review and meta-analysis. *The lancet Gastroenterology & hepatology* 2019; **4**(5): 389-98.

5. Rojas YAO, Cuellar CLV, Barrón KMA, Verdugo JPA, Miranda AL. Non-alcoholic Fatty Liver Disease prevalence in Latin America: A systematic review and meta-analysis. *Annals of hepatology* 2022: 100706.

6. Spearman CW, Afihene M, Betiku O, et al. Epidemiology, risk factors, social determinants of health, and current management for non-alcoholic fatty liver disease in sub-Saharan Africa. *The lancet Gastroenterology & hepatology* 2021; **6**(12): 1036-46.

7. Younossi ZM, Blissett D, Blissett R, et al. The economic and clinical burden of nonalcoholic fatty liver disease in the United States and Europe. *Hepatology (Baltimore, Md)* 2016; **64**(5): 1577-86.

8. Hagström H, Nasr P, Ekstedt M, et al. Health Care Costs of Patients With Biopsy-Confirmed Nonalcoholic Fatty Liver Disease Are Nearly Twice Those of Matched Controls. *Clinical gastroenterology and hepatology : the official clinical practice journal of the American Gastroenterological Association* 2020; **18**(7): 1592-9.e8.

9. Younossi ZM, Henry L. Economic and Quality-of-Life Implications of Non-Alcoholic Fatty Liver Disease. *PharmacoEconomics* 2015; **33**(12): 1245-53.

10. LaBrecque DR, Abbas Z, Anania F, et al. World Gastroenterology Organisation global guidelines: Nonalcoholic fatty liver disease and nonalcoholic steatohepatitis. *Journal of clinical gastroenterology* 2014; **48**(6): 467-73.

For peer review only - http://bmjopen.bmj.com/site/about/guidelines.xhtml

11. Chalasani N, Younossi Z, Lavine JE, et al. The diagnosis and management of nonalcoholic fatty liver disease: Practice guidance from the American Association for the Study of Liver Diseases. *Hepatology (Baltimore, Md)* 2018; **67**(1): 328-57.

12. Leoni S, Tovoli F, Napoli L, Serio I, Ferri S, Bolondi L. Current guidelines for the management of non-alcoholic fatty liver disease: A systematic review with comparative analysis. *World journal of gastroenterology* 2018; **24**(30): 3361-73.

13. Arab JP, Dirchwolf M, Álvares-da-Silva MR, et al. Latin American Association for the study of the liver (ALEH) practice guidance for the diagnosis and treatment of non-alcoholic fatty liver disease. *Annals of hepatology* 2020; **19**(6): 674-90.

14. European Association for the Study of the Liver (EASL), European Association for the Study of Diabetes (EASD), European Association for the Study of Obesity (EASO). EASL-EASD-EASO Clinical Practice Guidelines for the management of non-alcoholic fatty liver disease. *Diabetologia* 2016; **59**(6): 1121-40.

15. Mantovani A, Byrne CD, Targher G. Efficacy of peroxisome proliferator-activated receptor agonists, glucagon-like peptide-1 receptor agonists, or sodium-glucose cotransporter-2 inhibitors for treatment of non-alcoholic fatty liver disease: a systematic review. *The lancet Gastroenterology & hepatology* 2022; **7**(4): 367-78.

16. Ferguson D, Finck BN. Emerging therapeutic approaches for the treatment of NAFLD and type 2 diabetes mellitus. *Nature reviews Endocrinology* 2021; **17**(8): 484-95.

17. Fatima K, Moeed A, Waqar E, et al. Efficacy of statins in treatment and development of non-alcoholic fatty liver disease and steatohepatitis: A systematic review and meta-analysis. *Clinics and research in hepatology and gastroenterology* 2021; **46**(4): 101816.

18. Sarría-Santamera A, Orazumbekova B, Maulenkul T, Gaipov A, Atageldiyeva K. The Identification of Diabetes Mellitus Subtypes Applying Cluster Analysis Techniques: A Systematic Review. *International journal of environmental research and public health* 2020; **17**(24).

[dataset]19. Centers for Disease Control and Prevencion. National Center for Health Statistics. National Health and Nutrition Examination Survey. NHANES III (1988-1994). Available at: https://wwwn.cdc.gov/nchs/nhanes/nhanes3/default.aspx (Accessed May 18th, 2022).

For peer review only - http://bmjopen.bmj.com/site/about/guidelines.xhtml

20. National Health and Nutrition Examination Survey. 1988-1994 Data Documentation, Codebook, and Frequencies. Hepatic/Gallbladder Ultrasound and Hepatic Steatosis (HGUHS). Available at: https://wwwn.cdc.gov/nchs/data/nhanes3/34a/HGUHS.htm (Accessed May 18th, 2022).

21. Mahfood Haddad T, Hamdeh S, Kanmanthareddy A, Alla VM. Nonalcoholic fatty liver disease and the risk of clinical cardiovascular events: A systematic review and meta-analysis. *Diabetes & metabolic syndrome* 2017; **11 Suppl 1**: S209-s16.

22. Centers for Disease Control and Prevention. NHANES III - Laboratory Data File. Available at: https://wonder.cdc.gov/wonder/sci_data/surveys/hanes/hanes3/type_txt/lab.asp (Accessed May 18th 2022).

23. National Center for Health Statistics. 2019 Public - Use Linked Mortality Files. Available at: https://www.cdc.gov/nchs/data-linkage/mortality-public.htm (Accessed 19th May 2022).

24. Younossi Z, Anstee QM, Marietti M, et al. Global burden of NAFLD and NASH: trends, predictions, risk factors and prevention. *Nature reviews Gastroenterology & hepatology* 2018; **15**(1): 11-20.

25. Amanullah I, Khan YH, Anwar I, Gulzar A, Mallhi TH, Raja AA. Effect of vitamin E in non-alcoholic fatty liver disease: a systematic review and meta-analysis of randomised controlled trials. *Postgraduate medical journal* 2019; **95**(1129): 601-11.

26. European Association for the Study of the Liver (EASL), European Association for the Study of Diabetes (EASD), (EASO) EAftSoO. EASL-EASD-EASO Clinical Practice Guidelines for the management of non-alcoholic fatty liver disease. *Diabetologia* 2016; **59**(6): 1121-40.

27. Plaz Torres MC, Aghemo A, Lleo A, et al. Mediterranean Diet and NAFLD: What We Know and Questions That Still Need to Be Answered. *Nutrients* 2019; **11**(12).

28. Eslam M, Sanyal AJ, George J. MAFLD: A Consensus-Driven Proposed Nomenclature for Metabolic Associated Fatty Liver Disease. *Gastroenterology* 2020; **158**(7): 1999-2014.e1.

29. Golabi P, Paik JM, Arshad T, Younossi Y, Mishra A, Younossi ZM. Mortality of NAFLD According to the Body Composition and Presence of Metabolic Abnormalities. *Hepatology communications* 2020; **4**(8): 1136-48.

For peer review only

For peer review only - http://bmjopen.bmj.com/site/about/guidelines.xhtml

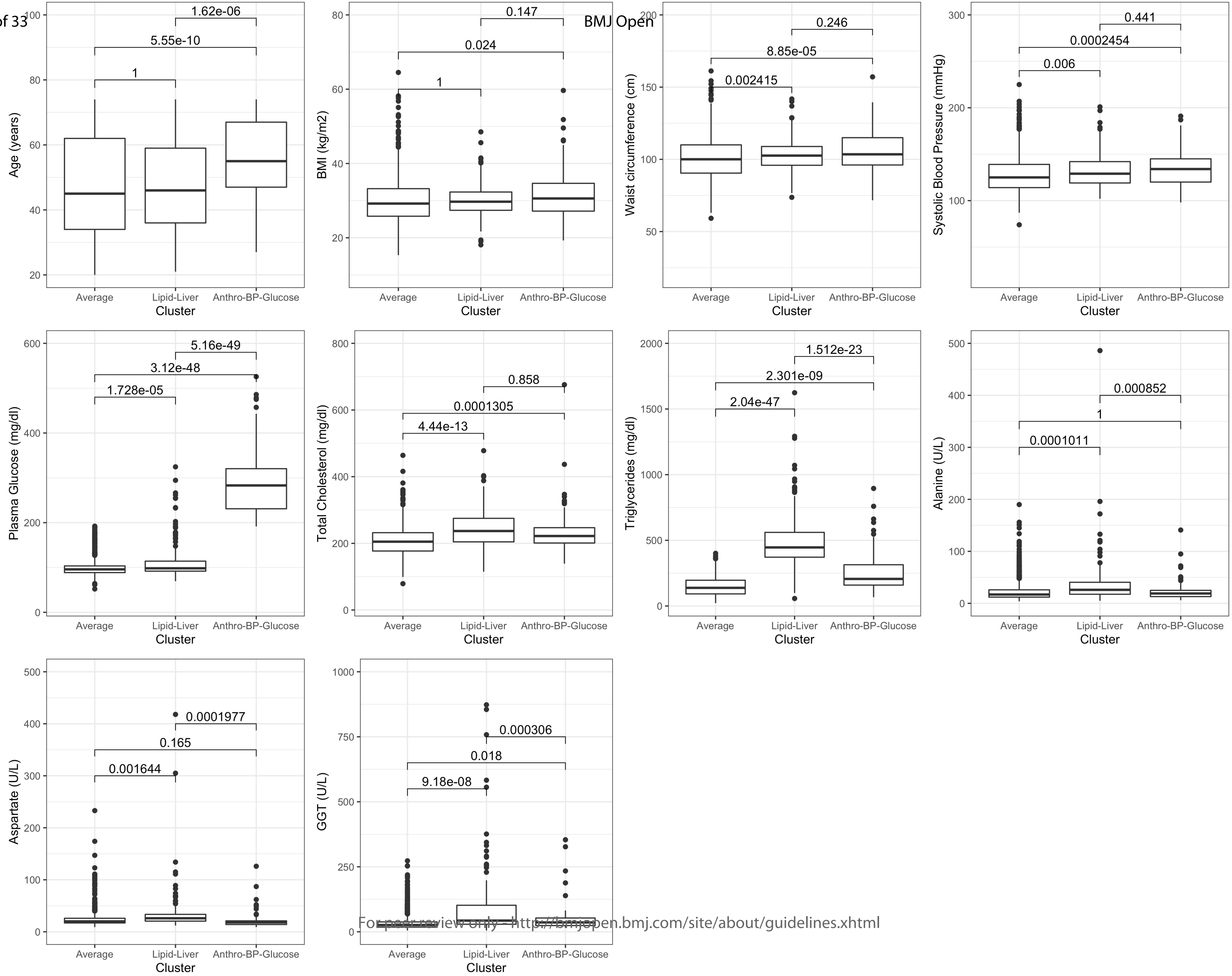
Age (years)
1.62e-06
5.55e-10
1
BMI (kg/m2)
0.147
0.024
1
Waist circumference (cm)
0.246
8.85e-05
0.002415
Systolic Blood Pressure (mmHg)
0.441
0.0002454
0.006
Plasma Glucose (mg/dl)
5.16e-49
3.12e-48
1.728e-05
Total Cholesterol (mg/dl)
0.858
0.0001305
4.44e-13
Triglycerides (mg/dl)
1.512e-23
2.301e-09
2.04e-47
Alanine (U/L)
0.000852
1
0.0001011
Aspartate (U/L)
0.0001977
0.165
0.001644
GGT (U/L)
0.000306
0.018
9.18e-08
Average
Lipid-Liver
Anthro-BP-Glucose
Cluster

SUPPLEMENTARY MATERIALS

Phenotypes of non-alcoholic fatty liver disease (NAFLD) and all-cause mortality: Unsupervised machine learning analysis with NHANES III

For peer review only

Table of Contents

Supplementary Methods 2
Data preparation 2
Study sample 2
Number of clusters 3
Supplementary Table 1. Pair-wise comparisons between clusters 7
Supplementary Figure 1. Survival curve by cluster 8
Supplementary Figure 2. Schoenfeld residuals for the crude Cox-proportional hazard model 9

For peer review only - http://bmjopen.bmj.com/site/about/guidelines.xhtml

Supplementary Methods

All the analyses were conducted with R and Python. The codes, either R scripts or Python Jupyter notebooks, are available as supplementary materials together with this manuscript.

Data preparation

We used individual-level data from the National Health and Nutrition Examination Survey (NHANES) III conducted between 1988-1994. All datasets were downloaded from the NHANES website on March 3rd, 2022; the mortality dataset was download on May 27th, 2022. The authors did not have privileged access. Data were pre-processed with R (NHANESiii_extraction.R). We used these datasets: adult.dat.txt (n=20,050); lab.sas.txt (n=29,314); exam.sas.txt (n=31,311); examdr.sas.txt (n=30,818); and HGUHS (n=14,797). The adult dataset was used as the prime; that is, the other datasets were merged to the adult dataset: adult + lab (n=20,050); + exam (n=20,050); + examdr (n=20,050); + HGUHS (n=20,050). We kept only the variables of interest, checked each variable, and recoded them as needed; for example, 9999 or equivalent values were set to missing, and 2 were coded to 0 when referred to 'no'. We merged the mortality dataset (n=33,994) with the pooled dataset and the sample size was kept at 20,050 (i.e., the pooled dataset was used as the prime) with 79 variables.

Study sample

We followed these criteria to select the study sample (Flowchart 1). First, we only included people in the age range from 20 to 74 (inclusive) years. Second, we only kept observations with hepatic imaging data. Third, we excluded people with positive evidence of Hepatitis B (HBsAG) or Hepatitis C (antiHCV). Fourth, we excluded people

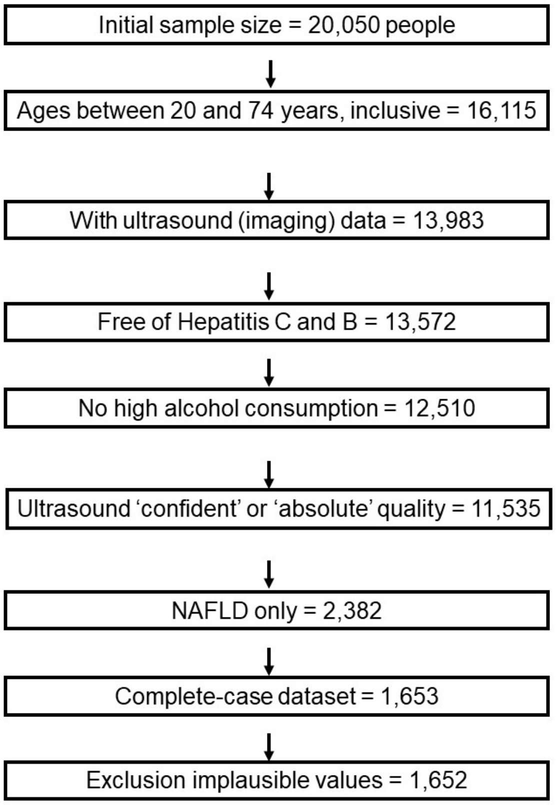

Flowchart 1. Study population.

For peer review only - http://bmjopen.bmj.com/site/about/guidelines.xhtml

with high alcohol consumption. We used two questions to define high alcohol consumption: i) *In the past 12 months, how many days of the year did you drink any alcoholic beverages?* and ii) *On the average, on the days that you drank alcohol, how many drinks did you have a day?* The answers to the second question were divided by 365.25 to compute the average number of drinks per day, which was then multiplied by the first question (how many days they drunk alcohol) to compute the number of daily alcohol drinks in the last year. For example, a person who drank alcoholic beverages 52 days in the last year, and on each occasion they drank three beverages, their daily consumption would be 52 x 3/365.25 = 0.43 alcoholic beverages per day in the last year. Men with more than two and women with more than one alcoholic beverages per day in the last year were excluded. Fifth, we only included observations which the hepatic imaging was deemed 'confident' or 'absolute'; this, to secure the highest quality of the outcome of interest (NAFLD); in addition, we only include people whose hepatic imaging revealed hepatic steatosis 'moderate-severe'. Sixth, we only kept the 10 predictors of interest and dropped all missing observations; in other words, we were to conduct a complete-case analysis. Seventh, we excluded observations outside the following plausibility ranges to secure high-quality data: BMI below and above 10 kg/m^2 and 80 kg/m^2; waist circumference below and above 30 cm and 200 cm; systolic blood pressure below and above 70 mmHg an 270 mmHg; fasting plasma glucose below and above 45 mg/dL and 540 mg/dL; total cholesterol below and above 20 mg/dL and 773 mg/dL; and triglycerides below and above 17 mg/dL and 1771 mg/dL. Finally, we included 1,652 observations in the analysis. For further details about this selection process please refer to the Jupyter notebook 1.Cleaning_data.ipynb.

Number of clusters

Selecting the *ideal* number of clusters in an unsupervised machine learning model is informed by both the data and expert knowledge. In here, we describe in detail the process we followed to reach the final number of clusters used in the analysis. Details about the analytical steps are presented in the Jupyter notebooks presented along the paper (2.Number_clusters.ipynb).

For peer review only - http://bmjopen.bmj.com/site/about/guidelines.xhtml

First, we displayed a dendrogram with Euclidean distances; this plot suggested there were five clusters. Of note, two of the six clusters grouped (very) few observations. Second, we displayed the Elbow plot (Figure 1) for one through 10 clusters. The ideal number of clusters would be that after which the Cost function does not change substantially and is the smallest. The table below shows the Cost function for each number of clusters, and the absolute arithmetic difference between two consecutive Cost functions. According to these figures (Table 1), the optimal number of clusters could be between five and six clusters; moreover, five clusters appeared to be *ideal* because the cost function was smallest and almost constant (~1.4) thereafter.

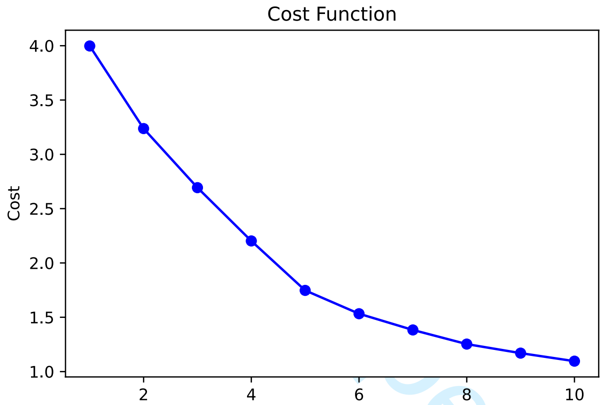

Figure 2. Elbow plot.

Table 1. Cost function for each number of cluster (i.e., elbow plot)

Number of clusters	Cost function	Difference with the immediate before
1	3.9975786924939500	
2	3.2371647353902400	-0.76
3	2.6931986798721200	-0.54
4	2.2026180284390100	-0.49
5	1.7472952094553600	-0.46
6	1.5326884453790900	-0.21
7	1.3831729448463400	-0.15
8	1.2530783891239300	-0.13
9	1.1698248951603600	-0.08
10	1.0958516180666800	-0.07

Third, we displayed the Silhouette plots and computed the Silhouette scores between two and 10 clusters (Table below). The number of clusters which Silhouette score is closest to 1 is preferred. According to these

For peer review only - http://bmjopen.bmj.com/site/about/guidelines.xhtml

figures (Table 2), two clusters would be *ideal* (i.e., highest Silhouette score), closely followed by three and four clusters.

Table 2. Silhouette score for each number of clusters.

Number of clusters	Silhouette scores
2	0.54608205593638
3	0.49123277407391
4	0.47822718686785
5	0.29615494816696
6	0.30743082083024
7	0.24963726715962
8	0.26441204421507
9	0.25048383421862
10	0.25036003409133

For peer review only

Fourth, we calculated the Jaccard index for three, four and five clusters (Table 3). So far, three, four and five clusters appear to be the best options. Jaccard scores ≥0.80 suggest good reproducibility of the cluster and would thus be preferred. According to these figures (Table below), three clusters had the highest Jaccard scores, all of which were ≥0.88. The Jaccard analysis was conducted in R (2.1.Jaccard.R).

Table 3. Jaccard score for each number of clusters from three to five clusters.

	**1 cluster**	**2 clusters**	**3 clusters**		
Jaccard bootstrap	0.8853153	0.9666082	0.9000518		
Dissolved	284	0	309		
Recovered	4402	4826	4579		
	**1 cluster**	**2 clusters**	**3 clusters**	**4 clusters**	
Jaccard bootstrap	0.5858137	0.8741936	0.5433940	0.6849515	

For peer review only - http://bmjopen.bmj.com/site/about/guidelines.xhtml

Dissolved	2564	0	2801	720	
Recovered	1728	4705	1279	1691	
	**1 cluster**	**2 clusters**	**3 clusters**	**4 clusters**	**5 clusters**
Jaccard bootstrap	0.7489208	0.7477933	0.5354848	0.5248099	0.6352783
Dissolved	1244	17	2839	2834	1918
Recovered	2942	2231	915	1139	1905

Fifth, exploratory analyses with five and six clusters showed that some clusters had (very) few observations and there were not unique profiles to characterize these clusters. Having four clusters was later discarded because of the Jaccard scores (see Table above). In conclusion, we chose three clusters as the *ideal* number of clusters for the analysis, which is also supported by the Jaccard score and (rather) consistent with the Elbow plot (Figure 1) and the Silhouette score (Table 2).

Of note, in exploratory analyses, we also tried selecting the number of clusters based on other algorithms and the results were consistent with those herein summarized; the algorithms we tried were: Balanced Iterative Reducing and Clustering using Hierarchies (BIRCH); spectral; and agglomerative.

For peer review only

Supplementary Table 1. Pair-wise comparisons between clusters.

Predictor	Grupo 1	Grupo 2	Sample in Group 1	Sample in Group 2	Statistic	Degrees of freedom	p-value	Bonferroni-adjusted p-value
Age	Average	Lipid-Liver	1376	171	-0.4341	228.8684	6.650E-01	1.000E+00
	Average	Anthro-BP-Glucose	1376	105	-6.9071	132.8967	0.000E+00	0.000E+00
	Lipid-Liver	Anthro-BP-Glucose	171	105	-5.1494	243.0031	0.000E+00	0.000E+00
Body Mass Index	Average	Lipid-Liver	1376	171	-0.8983	254.9349	3.700E-01	1.000E+00
	Average	Anthro-BP-Glucose	1376	105	-2.7146	118.85	8.000E-03	2.400E-02
	Lipid-Liver	Anthro-BP-Glucose	171	105	-1.9792	167.1476	4.900E-02	1.470E-01
Waist Circumference	Average	Lipid-Liver	1376	171	-3.3914	256.2415	8.000E-04	2.400E-03
	Average	Anthro-BP-Glucose	1376	105	-4.3361	125.4833	0.000E+00	1.000E-04
	Lipid-Liver	Anthro-BP-Glucose	171	105	-1.7477	190.4266	8.200E-02	2.460E-01
Systolic Blood Pressure	Average	Lipid-Liver	1376	171	-3.0838	221.5132	2.000E-03	6.000E-03
	Average	Anthro-BP-Glucose	1376	105	-4.0781	120.6513	1.000E-04	2.000E-04
	Lipid-Liver	Anthro-BP-Glucose	171	105	-1.4565	209.4584	1.470E-01	4.410E-01
Plasma Glucose	Average	Lipid-Liver	1376	171	-4.6764	177.7001	0.000E+00	0.000E+00
	Average	Anthro-BP-Glucose	1376	105	-26.7622	105.0216	0.000E+00	0.000E+00
	Lipid-Liver	Anthro-BP-Glucose	171	105	-22.3014	150.0289	0.000E+00	0.000E+00
Total Cholesterol	Average	Lipid-Liver	1376	171	-7.9482	195.336	0.000E+00	0.000E+00
	Average	Anthro-BP-Glucose	1376	105	-4.2568	111.1312	0.000E+00	1.000E-04
	Lipid-Liver	Anthro-BP-Glucose	171	105	1.0709	197.489	2.860E-01	8.580E-01
Triglycerides	Average	Lipid-Liver	1376	171	-20.3273	174.601	0.000E+00	0.000E+00
	Average	Anthro-BP-Glucose	1376	105	-6.7507	107.88	0.000E+00	0.000E+00
	Lipid-Liver	Anthro-BP-Glucose	171	105	11.1491	272.009	0.000E+00	0.000E+00
Aspartate	Average	Lipid-Liver	1376	171	-3.521	175.1681	5.000E-04	1.600E-03
	Average	Anthro-BP-Glucose	1376	105	1.9415	118.8264	5.500E-02	1.650E-01
	Lipid-Liver	Anthro-BP-Glucose	171	105	4.0638	234.143	1.000E-04	2.000E-04
Alanine	Average	Lipid-Liver	1376	171	-4.2566	176.3829	0.000E+00	1.000E-04
	Average	Anthro-BP-Glucose	1376	105	-0.2754	118.8691	7.840E-01	1.000E+00
	Lipid-Liver	Anthro-BP-Glucose	171	105	3.683	244.7561	3.000E-04	9.000E-04
Glutamyl	Average	Lipid-Liver	1376	171	-5.8033	171.8756	0.000E+00	0.000E+00
	Average	Anthro-BP-Glucose	1376	105	-2.8266	108.7452	6.000E-03	1.800E-02
	Lipid-Liver	Anthro-BP-Glucose	171	105	3.9527	239.6927	1.000E-04	3.000E-04

For peer review only

For peer review only - http://bmjopen.bmj.com/site/about/guidelines.xhtml

Supplementary Figure 1. Survival curve by cluster.

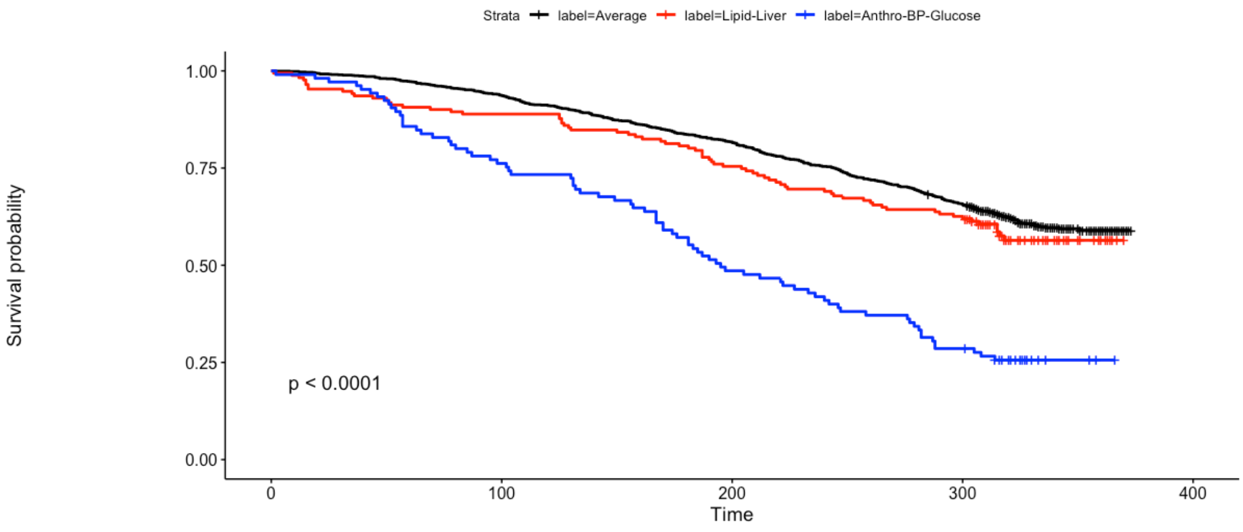

Number at risk

Strata	0	100	200	300	400
label=Average	1375	1289	1124	904	0
label=Lipid-Liver	171	152	129	107	0
label=Anthro-BP-Glucose	105	80	51	30	0

Time

For peer review only - http://bmjopen.bmj.com/site/about/guidelines.xhtml

Supplementary Figure 2. Schoenfeld residuals for the crude Cox-proportional hazard model.

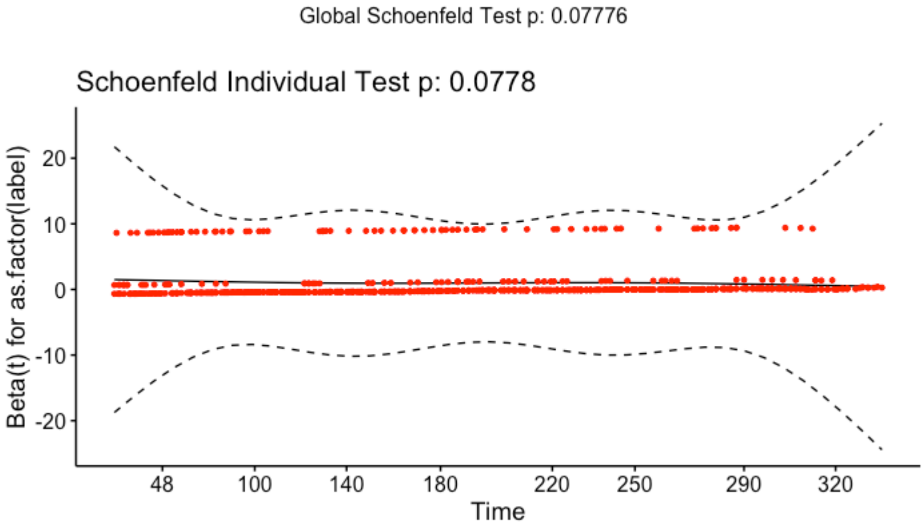

For peer review only
